# Regulation of Gene Expression in Plants through miRNA Inactivation

**DOI:** 10.1371/journal.pone.0021330

**Published:** 2011-06-23

**Authors:** Sergey Ivashuta, Isaac R. Banks, B. Elizabeth Wiggins, Yuanji Zhang, Todd E. Ziegler, James K. Roberts, Gregory R. Heck

**Affiliations:** Monsanto Company, Chesterfield, Missouri, United States of America; Emory Unviersity, United States of America

## Abstract

Eukaryotic organisms possess a complex RNA-directed gene expression regulatory network allowing the production of unique gene expression patterns. A recent addition to the repertoire of RNA-based gene regulation is miRNA target decoys, endogenous RNA that can negatively regulate miRNA activity. miRNA decoys have been shown to be a valuable tool for understanding the function of several miRNA families in plants and invertebrates. Engineering and precise manipulation of an endogenous RNA regulatory network through modification of miRNA activity also affords a significant opportunity to achieve a desired outcome of enhanced plant development or response to environmental stresses. Here we report that expression of miRNA decoys as single or heteromeric non-cleavable microRNA (miRNA) sites embedded in either non-protein-coding or within the 3′ untranslated region of protein-coding transcripts can regulate the expression of one or more miRNA targets. By altering the sequence of the miRNA decoy sites, we were able to attenuate miRNA inactivation, which allowed for fine regulation of native miRNA targets and the production of a desirable range of plant phenotypes. Thus, our results demonstrate miRNA decoys are a flexible and robust tool, not only for studying miRNA function, but also for targeted engineering of gene expression in plants. Computational analysis of the *Arabidopsis* transcriptome revealed a number of potential miRNA decoys, suggesting that endogenous decoys may have an important role in natural modulation of expression in plants.

## Introduction

RNA-based gene regulation has been identified as one of the major conserved mechanisms in eukaryotes [1] and represents an attractive modality for trait engineering in plants. MicroRNAs (miRNAs) are small RNAs that regulate gene expression by targeting one or more sequences of high complementarity in plants [2]. Many plant miRNAs change their expression during development or in response to environmental challenges, and regulate protein-coding genes involved in development, stress response and nutrient transport [2], [3]. According to miRBase database Release 15.0 (http://microrna.sanger.ac.uk/), there are about 1000 registered miRNAs in plants that are predicted to regulate hundreds of genes, many of which are transcription factors that in turn regulate multiple genes [2], [4]. Typically, plant miRNAs repress their targets through cleavage at nucleotide positions 10–11 relative to the 5′end of the complementary miRNA sequence [5], however, translational regulation has also been recently demonstrated to be widespread in plants [6]. Functional non-coding RNAs with natural non-cleavable miRNA sites have also recently been described in *Arabidopsis*. These transcripts are able to bind their complementary miRNAs and, in this context, have a role in trans-acting siRNA biogenesis [7] as well as the regulation of miR399 activity in *Arabidopsis* by functioning as miRNA decoys [8]. Over-expression of miRNA decoys with non-cleavable miRNA sites, termed miRNA mimics in plants [8], and microRNA sponges in animals [9],was suggested to be a useful tool for understanding the function of miRNA families in plants and invertebrates. While an optimized miRNA decoy approach has become a very popular tool for regulating miRNAs in various animal applications, and is a proposed natural mechanism of animal gene regulation (reviewed in [10]), there are a limited number of publications [8], [11] demonstrating the utility of this approach in plants.

We propose that using non-cleavable miRNA sites for the regulation of endogenous miRNAs can enhance our ability to manipulate complex traits in plants. However, the flexibility and robustness of the miRNA decoy approach has not yet been rigorously examined. In addition, the utility of this approach for crop improvement can be restricted by such challenges as the limited ability to control the degree of miRNA inactivation, the need for specific inactivation of individual members of large miRNA families, as well as the lack of an appropriate strategy for non-cleavable miRNA target site expression in plants compatible with commonly used transgenic methods. Our results suggest that the expression of single or heteromeric non-cleavable miRNA decoys, embedded in either non-protein-coding or protein-coding transcripts, can provide a tool for synchronized regulation of expression of miRNA targets. Importantly, the level of miRNA inactivation can be regulated by modifying the miRNA decoy sequence, thus achieving a desirable range of phenotypic readouts. In addition, our computational analysis indentified a number of putative miRNA decoys in *Arabidopsis*, suggesting that miRNA decoy-based regulation may be relatively widespread in plants.

## Results

### Artificial miRNA decoys expressed from different RNA scaffolds result in miRNA inactivation

Initially, two types of synthetic miRNA decoys were tested: first, miRNA decoys with three nucleotide insertions between the nucleotide positions corresponding to nucleotides 10–11 of the miRNA, similar to that described previously [8], thus creating a bulge upon miRNA annealing; and second, a miRNA decoy with two mismatches corresponding to positions 10–11 of the miRNA sequence (Fig. S1A). The decoy sites were placed within a 640 nt non-coding transcript cloned from maize (*Zea mays* L.), named Zma-miR399 mimic-like (MIM), replacing the endogenous non-cleavable miR399 site (Table S1). Based on sequence conservation of the 24 nucleotide Zma-miR399 MIM site to the Ath-miR399 MIM site within the *IPS1* non-coding RNA in *Arabidopsis* [8], we anticipated that the maize Zma-miR399 MIM transcript would function similarly to Ath *IPS1* and could serve as a scaffold for new decoy testing. We predicted that a target miRNA would bind to the synthetic miRNA decoy when both miRNA and miRNA decoy were expressed in plants, resulting in sequestration of the miRNA and stabilization of a reporter gene harboring a corresponding miRNA target site (Fig. S1B). Results of initial experiments indicated that this was indeed the case. Both bulged and mismatched miRNA decoys can specifically bind and sequester several corresponding endogenous and synthetic miRNAs (Table S1) when co-expressed in *Nicotiana benthamiana*, as measured by expression of a reporter gene.

**Figure 1:**
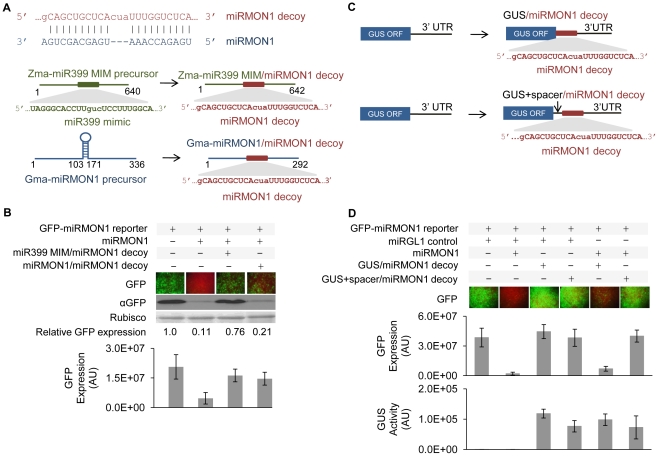
Effect of different RNA scaffolds on miRNA decoy efficacy. (**A**) miRMON1 bulge decoy sequence and diagrams of miRMON1 decoy embedded in various backbone configurations: miRMON1 decoys in maize non-coding RNA and soybean miRMON1 precursor. (**B**) Expression of miRMON1-targeted GFP reporter co-expressed with miRNA decoys in different scaffolds in *N. benthamiana* leaves. (**C**) Diagram of miRMON1 decoys in the 3′ UTR of the GUS coding transcript, with and without a 75 nt spacer after the open reading frame. (**D**) Expression of miRMON1-targeted GFP reporter co-expressed with decoys in a GUS protein-coding cassette in *N. benthamiana* leaves. GFP expression was measured by fluorescence microscopy and Western blot. The Rubisco band visualized by Ponceau staining shows the loading control. Relative GFP protein expression normalized to Rubisco is indicated. GUS expression was measured by the 4-methylumbelliferone assay. Leaves were co-transformed with one part GFP reporter, 5 parts miRMON1, and 10 parts miRNA decoy. *Agrobacterium* transformed with an empty vector was added to achieve a total OD_600_ = 1 of *Agrobacterium* for each transformation.

We evaluated the adaptability of this miRNA decoy approach to transcript backbones other than the miRNA mimic-like backbone using the miRMON1/miRMON1 decoy pair as an example (Fig. 1A). miRMON1 was previously identified in soybean (*Glycine max* L.), data not shown, and is absent in both *Arabidopsis* and *N. benthamiana*. The Gma-miRMON1 precursor sequence was modified by replacing the stem-loop structure with a synthetic decoy site partially complementary to the mature Gma-miRMON1, thus converting a native Gma-miRMON1 precursor into an engineered miRMON1 decoy transcript (Fig. 1A, Table S1). Co-expression of miRMON1 with the engineered miRMON1 decoy and a GFP-miRMON1 reporter in *N. benthamiana* resulted in stabilization of the GFP-miRMON1 transcript and increased GFP activity (Fig. 1B), similar to our observations when using the Zma-miR399 MIM backbone, indicating that the modified soybean miRNA precursor can serve as a scaffold for engineered miRNA decoys. However, the relative activity of the decoy embedded in the miRMON1 precursor was lower as compared with the miR399 MIM backbone.

The above data suggest that miRNA decoys are functional when expressed as part of various non-coding transcripts, however, we also wanted to determine if a miRNA decoy could effectively sequester miRNAs when expressed as part of a protein-coding transcript. This approach would be beneficial in cases where the expression of a transgenic protein and a miRNA decoy need to be synchronized, or as a strategy to reduce the number of redundant transgenic elements expressed in plants. Two constructs were created in which the miRMON1 decoy site was either introduced immediately after the stop codon of a β-glucuronidase (GUS) open reading frame (ORF), or separated from the GUS ORF by a synthetic 75 bp spacer in the 3′ untranslated region (UTR, Fig. 1C). We hypothesized that scanning ribosomes might dissociate the miRNA/miRNA decoy complex if adjacent to the stop codon. It has been shown that ribosome progression along mRNA during translation would interfere with the ability of the miRNA to attach to its target site within the coding region in mammalian cells [12]. Indeed, the miRNA decoy site adjacent to the GUS ORF was less efficient in inactivating miRMON1, resulting in low expression of the GFP reporter as compared to the miRMON1 decoy site separated by a 75 bp spacer from the GUS ORF (Fig. 1D). Thus, this result demonstrates that miRNA decoys can inhibit miRNA activity when embedded in UTRs of protein–coding transcripts, providing an opportunity to express proteins and inactivate miRNAs using a single transgenic cassette. To investigate if an engineered miRNA decoy resident in the 3′ UTR of a protein–coding gene interferes with protein translation, we measured GUS activity of GUS/miRMON1 decoy constructs expressed in plants. We did not observe significant reduction of GUS activity in decoy constructs (Fig. 1D). Overall, these results suggest that miRNA decoys expressed in multiple transcript contexts can inactivate miRNAs *in planta* and lead to reduced function of the corresponding miRNA.

### Inactivation of multiple miRNAs using a heteromeric miRNA decoy cassette

**Figure 2:**
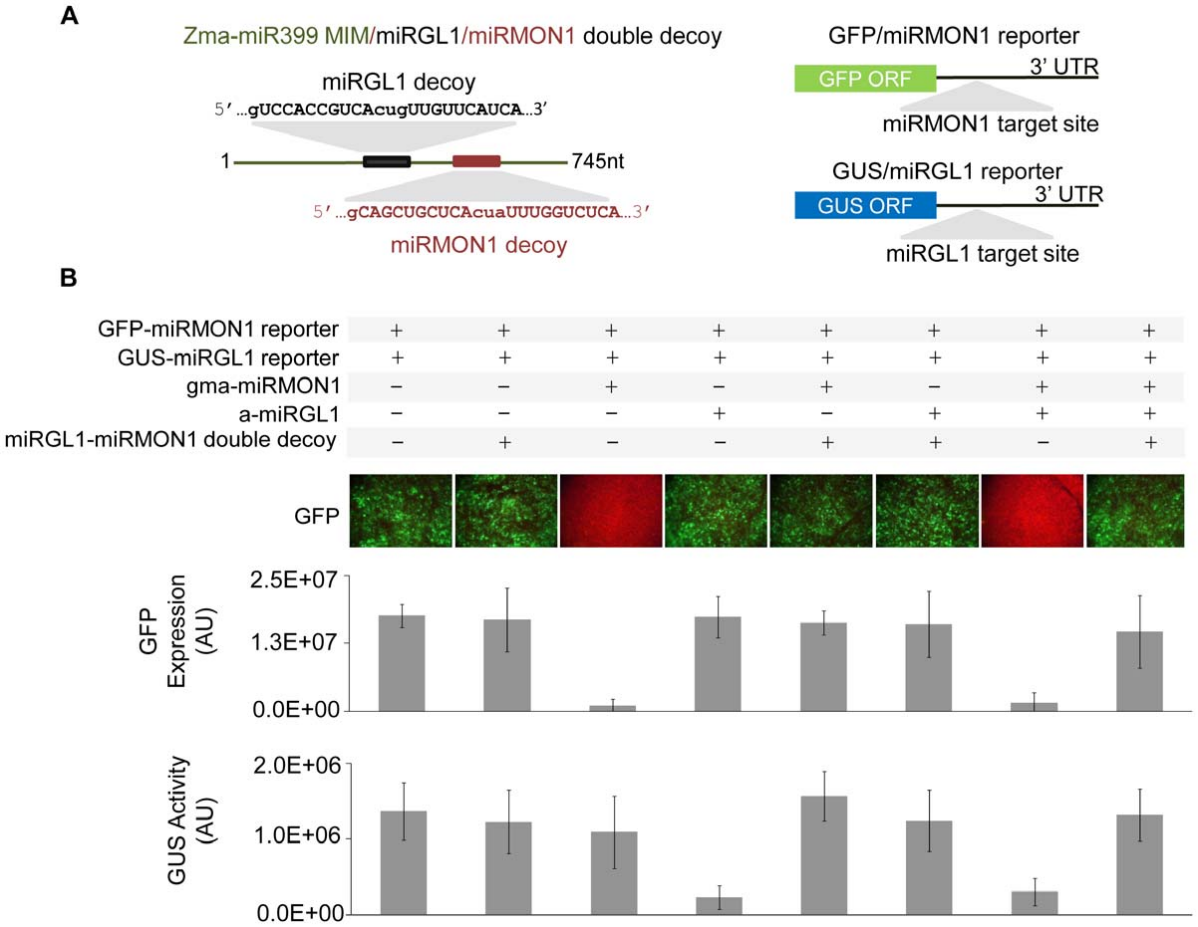
Inactivation of multiple miRNAs using a heteromeric miRNA decoy cassette. (**A**) Diagram of heterodimeric miRNA decoy transcript designed to bind both miRMON1 and a-miRGL1. (**B**) Quantitative analysis of micrographs of miRMON1-targeted GFP reporter intensity and of miRGL1-targeted GUS reporter in *N. benthamiana* leaves. Leaves were co-transformed with one part GFP and/or one part GUS reporter, 5 parts miRMON1 and/or 5 parts miRGL1, and ten parts miRNA decoy. *Agrobacterium* transformed with a control vector was added to achieve a total OD_600_ = 1 of *Agrobacterium* for each transformation. GFP intensity values are averages of six individual transformations. Tissue from the seven individual transformations per inoculum mix was used to measure GUS activity.

To test if we could inactivate more than one miRNA with a single transgenic transcript harboring multiple miRNA decoy sites, we created a construct carrying Gma-miRMON1 and artificial miRGL1 (a-miRGL1) decoy sites, both embedded in the Zma-miR399 MIM backbone (Fig. 2A). This was co-transformed with Gma-miRMON1 and a-miRGL1, along with GFP and GUS reporter constructs carrying Gma-miRMON1 and a-miRGL1 target sites in their 3′ UTRs, respectively. Results indicate that both Gma-miRMON1 and a-miRGL1 can be efficiently sequestered by a single heterodimeric miRNA decoy, as shown by an increase in reporter gene expression specifically in the presence of the miRNA decoys (Fig. 2B). The ability to inactivate a selected set of miRNAs allows regulation of multiple endogenous miRNA targets coding for genes involved in related or unrelated processes and provides an opportunity for complex trait engineering in plants.

### Tuning miRNA activity by variation of miRNA decoy expression and sequence composition

**Figure 3:**
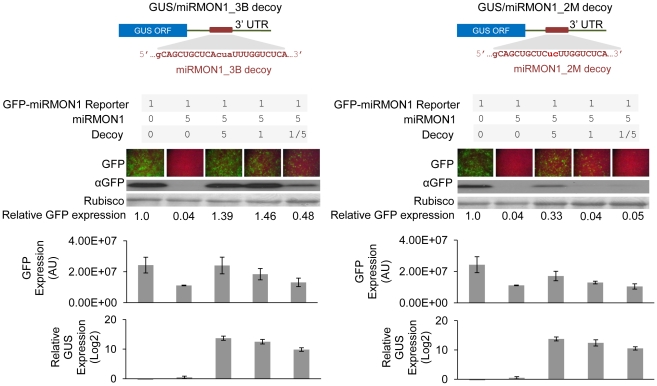
Effect of stoichiometry on activity of two miRMON1 decoys (bulged, miRMON1_3 B decoy, and mismatched, miRMON1_2 M decoy). Diagram of miRMON1_3B and 2M decoys embedded in the 3′ UTR of a GUS coding transcript and expression of miRMON1-targeted GFP reporter co-expressed with various concentrations of miRNA decoys in *N. benthamiana* leaves. Leaves were co-transformed with one part GFP reporter, 5 parts miRMON1, and variable parts miRNA decoy. *Agrobacterium* transformed with an empty vector was added to achieve a total OD_600_ = 1 of *Agrobacterium* for each transformation. GFP expression was measured by fluorescence microscopy and protein blot. The Rubisco band visualized by Ponceau staining shows the loading control. Relative GFP expression normalized to Rubisco and relative GUS expression as measured by qRT-PCR are indicated.

**Figure 4:**
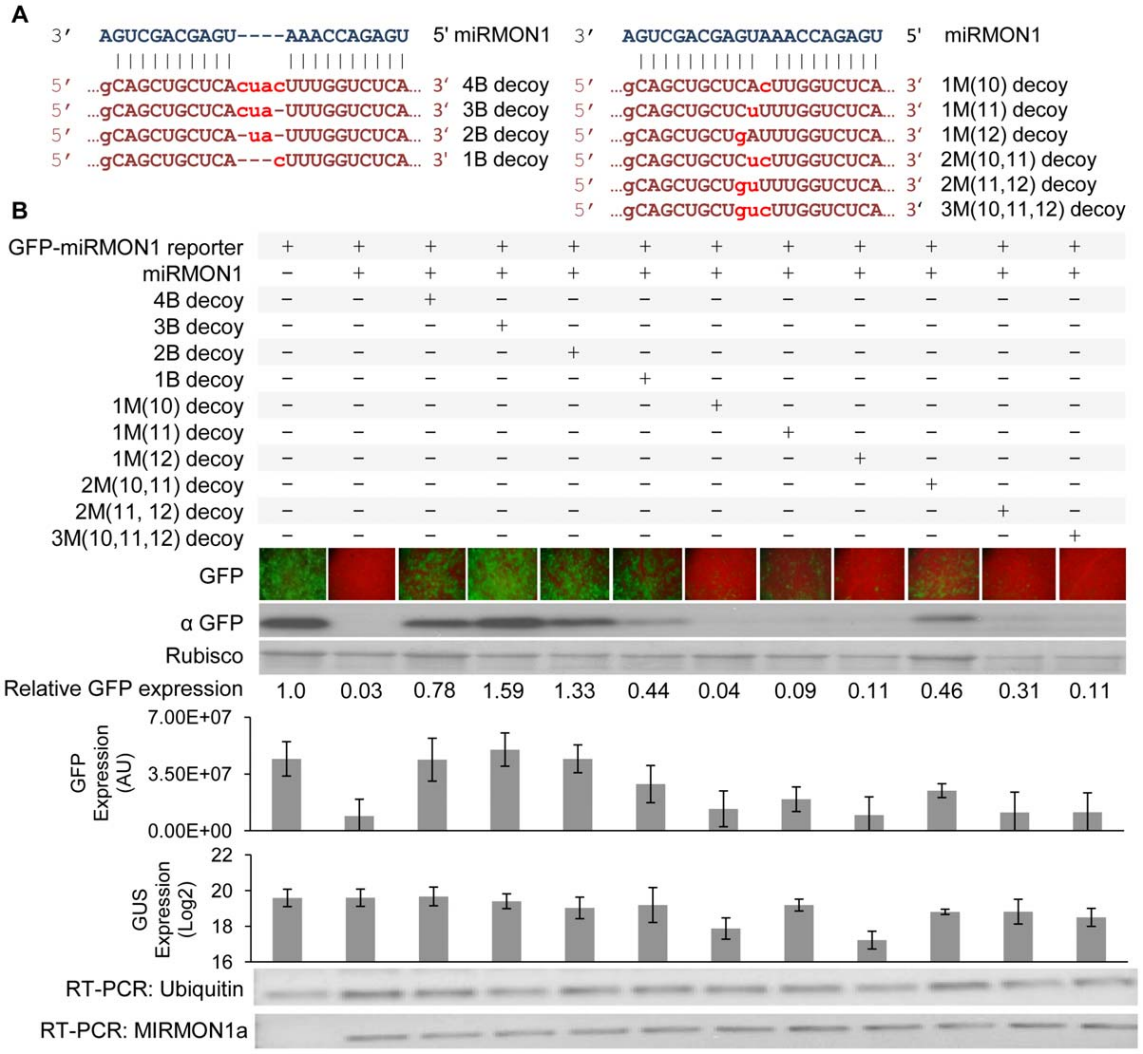
Effect of miRNA decoy structure on efficacy. (**A**) Sequence of miRMON1 decoys with bulged (4B, 3B, 2B, 1B) or mismatched (1M, 2M, 3M) structures at various positions between 10 and 12 of the miRNA (position of mutation in parentheses). (**B**) Expression of miRMON1-targeted GFP reporter co-expressed with various miRNA decoys in *N. benthamiana* leaves measured by fluorescence microscopy and Western blot. Leaves were co-transformed with one part GFP reporter, 5 parts miRMON1, and 10 parts miRNA decoy. *Agrobacterium* transformed with an empty vector was added to achieve a total OD_600_ = 1 of *Agrobacterium* for each transformation. The Rubisco band visualized by Ponceau staining shows the loading control. Relative GFP expression normalized to Rubisco is indicated. Relative GUS expression as measured by qRT-PCR indicates decoy expression, and miRMON1 expression was determined by semi-quantitative RT-PCR.

The over-expression of miRNA-resistant versions of endogenous miRNA targets, in which miRNA-mediated regulation is disrupted by converting target sites into non-cleavable miRNA resistant sites, can often lead to dramatic phenotypes and abnormal development, especially when modifying targets regulated by conserved miRNAs [2], [8]. While strong phenotypes are useful in understanding miRNA function in plants, such pronounced effects could reduce the value of miRNA decoy-based approaches for crop improvement. In many cases, fine-tuning miRNA activity rather than significant inactivation is necessary to prevent deleterious effects on plant development. The relative abundance, or expression level, of the miRNA decoy and target miRNA is obviously an important factor one must consider in order to achieve a desired outcome. To better understand the relative stoichiometric parameters involved in miRNA/miRNA decoy interaction, levels of miRMON1 decoys (bulged or mismatched, Fig. 3) were varied to observe the impact on miRMON1 activity. An inverse correlation was noted between miRMON1 decoy abundance and miRMON1 activity for both bulged and mismatched decoys, as measured by qRT-PCR of GUS and the expression level of GFP (Fig. 3). While the dilution of the bulged decoy is not directly comparable to the dilution of the mismatched decoy, the inverse correlation trend is noticeable. These results suggest that the degree of miRNA inactivation can be controlled to some extent by regulating the expression of miRNA decoy transgenic cassettes, for example, by selecting an appropriate promoter. In addition, we also noted a difference in efficiency of restoring GFP reporter expression between bulge and mismatch miRNA decoys (Fig. 3), despite similar miRNA decoy expression levels. To systematically investigate the efficiency of various miRNA decoys, we constructed a series of miRMON1 decoys, separated from the GUS ORF by a synthetic 75 bp spacer in the 3′UTR, with varying numbers of mismatches or bulges relative to the miRMON1 sequence (Fig. 4A). When expressed at similar levels, miRMON1 bulge decoys with one to four nucleotide insertions had the greatest ability to inactivate miRMON1. A bulge decoy with 5 insertions also inactivated miRMON1, though to a lesser extent, and bulge decoys with 6 or 7 insertions had low relative activity (Fig. S2). miRMON1 decoys with mismatches had an intermediate or low relative activity, depending on the number and position of mismatches to the miRNA (Fig. 4B). Decoy expression was measured by qRT-PCR of GUS, and was shown to be lower for decoys with a single mismatch at either position 10 or 12, presumably due to cleavage by miRMON1. miRMON1 precursor expression was measured by semi-quantitative RT-PCR and was uniform among treatments. The range of activity for miRNA decoys observed in our experiments provides an opportunity to systematically titrate endogenous miRNA activity for achieving a desired level of miRNA target up-regulation.

### Tuning plant phenotype by varying miRNA decoy sequence composition

**Figure 5:**
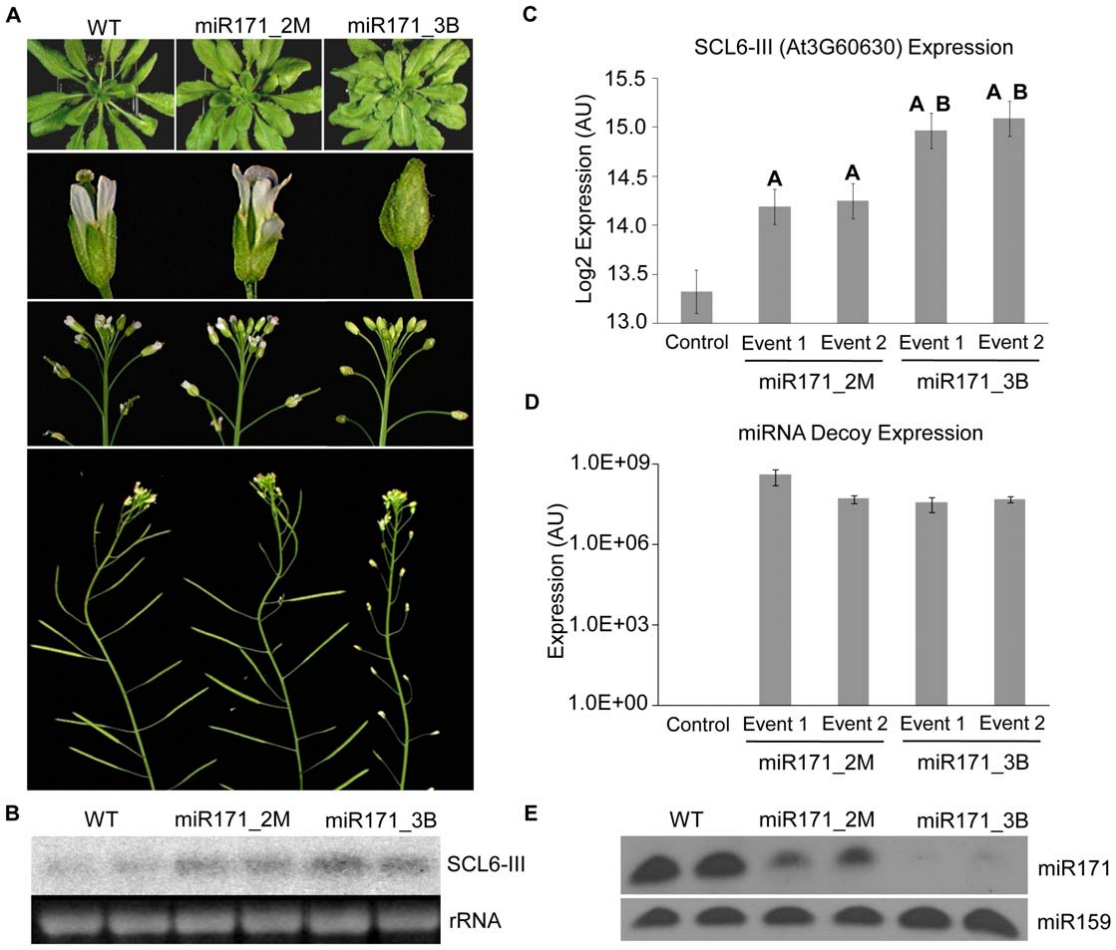
Phenotypic and expression analysis of transgenic *Arabidopsis* expressing miR171_2M and miR171_3B decoys. (**A**) Leaf and flower phenotypes of plants expressing miR171 decoys as compared to wild-type (WT). Expression of *SCL6-III* in miR171_2M and miR171_3B decoy plants verified by (**B**) northern blot analysis in leaves from 4 week old plants, with two independent events per group shown and (**C**) qRT-PCR. Log2-transformed expression values are plotted; error bar is plotted with +/− one standard error estimated from a two-way ANOVA. An “A” indicates significantly higher expression compared with control. miR171_3B events with significantly higher expression compared with miR171_2M events are indicated with a “B”. All statistical analysis can be found in Tables S2 and S3, online. (**D**) The corresponding expression of the decoy transgene for each event, using qRT-PCR. (**E**) Detection of mature miR171 via northern blot. miR159 is used as the control.

**Figure 6:**
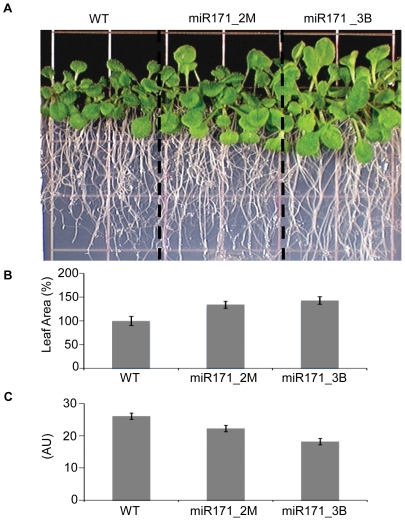
Characteristics of transgenic *Arabidopsis* over-expressing miR171_2M and miR171_3B decoys. (**A**) Wild type and transgenic plants (T3 generation) expressing miR171_2M and miR171_3B decoys grown on vertical agar plates with M&S media at 25°C and 16/8 hours day/night. (**B**) Rosette leaf area of wild type and transgenic plants expressing miR171_2M and miR171_3B decoys. Leaves were detached from plants and leaf area measured. Six three week old plants were used per measurement. (**C**) Chlorophyll content of miR171 decoy plants. Chlorophyll content of 3 rosette leaves of 12 plants per event was measured using Minolta SPAD 502 meter.

To assess the level of endogenous miRNA target regulation by bulge and mismatch decoys, we designed two miRNA decoy constructs to inactivate miR171a in *Arabidopsis*. In *Arabidopsis*, Ath-miR171 targets multiple SCARECROW-like proteins (SCL). This family of transcription factors has been implicated in radial patterning in roots, as well as gibberellin and light signaling pathways [13], [14]. Engineered miR171 decoys, with two mismatches (miR171_2M) at positions 10 and 11 or with a three nucleotide insertion (miR171_3B) between positions 10 and 11 were expressed using the Zma-miR399 MIM backbone in *Arabidopsis* under a constitutive 35S promoter. Approximately half of 26 independent transgenic T1 events expressing the miR171_2M decoy, and the majority of 16 T1 events expressing the miR171_3B decoy displayed a modified phenotype from wild-type, while the phenotypic spectrum varied significantly. Phenotypic evaluation of T2 and T3 progeny of two miR171_2M and two miR171_3B events displaying the strongest phenotypes and similarly high levels of transgene expression consistently revealed a milder phenotype in miR171_2M plants compared with miR171_3B plants. Both miR171_2M and miR171_3B plants had larger leaves, increased rosette leaf area, pale-green leaf color, and larger root systems when grown in soil (T2 generation) or on vertical agar plates (T3 generation) compared with wild-type plants, though miR171_2M plants showed intermediate phenotype severity relative to miR171_3B and wild-type (Fig. 5A, Fig. 6A–C). Both miR171_2M and miR171_3B plants also showed modified leaf angle growth when compared with control plants. In response to limited light, *Arabidopsis* controls leaf position through phototropic movement in order to optimize photosynthetic activity [15], [16]. miR171_2M and miR171_3B plants have limited upward movement of rosette leaves resulting in a flat rosette, with miR171_2M plants showing an intermediate rosette leaf angle relative to miR171_3B and wild-type (Fig. S3). A more dramatic difference between miR171_2M and miR171_3B plants was observed in floral development. miR171_3B plants exhibited a closed bud phenotype, likely due to altered sepal development, that resulted in the carpel bending inside of the closed flower (Fig. S4). This aberrant floral morphology resulted in significantly decreased seed set in miR171_3B plants due to reduced pollination of the closed flowers (Fig. 5A). When sepals were removed or pulled back from miR171_3B flowers, plants produced fertile siliques. The pale-green color of leaves and the flower phenotype observed in our experiment are consistent with recently published data and, as suggested, the result of miR171a inactivation in *Arabidopis* [11]. Phenotypic differences between *Arabidopsis* plants expressing miR171_2M and miR171_3B decoys were analogous to that of the weaker impact of the 2M decoy versus 3B decoy on miRMON1 activity in *N. benthamiana* (Fig. 3, Fig. 4B). Northern and qRT-PCR expression analysis indicated increased levels of miR171 target *SCL6-III* in miR171_3B decoy plants when compared to miR171_2M plants with similar levels of transgenic miRNA decoy expression (Fig. 5B–D, Table S2 and S3). Consistent with data presented previously [11], the abundance of mature miR171 detected via northern was significantly reduced in events expressing miR171 decoys, relative to WT plants. Interestingly, miR171 was shown to be far more depleted in events expressing the miR171_3B decoy than in events expressing the miR171_2M decoy, an inverse correlation to the corresponding miR171 target expression data (Fig. 5C, E). These data suggest that a combination of appropriate expression parameters with miRNA decoy sequence variation may provide the flexibility required for utilization of the miRNA decoy approach for crop improvement.

### Computational prediction of natural miRNA decoys

**Figure 7:**
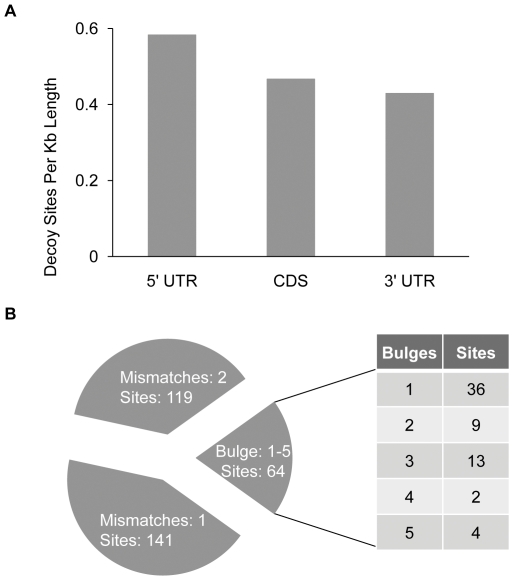
Computational prediction of endogenous miRNA decoys in Arabidopsis. (**A**) Distribution of predicted decoy sites in 5′ UTR, CDS and 3′ UTR of 286 protein-coding transcripts. If a decoy site spans two regions, i.e., 5′ UTR – CDS or CDS – 3′ UTR, the decoy site is assigned to the region in which the majority of the site is contained. The length of the 5′ UTR, CDS, and 3′ UTR of 286 transcripts is tallied respectively, and then the number of decoy sites for each feature is normalized to 1 kb sequence length. (**B**) Predicted decoy sites are classified into the ‘bulge’ type if there are bulges corresponding to miRNA bases 10–11, otherwise, they are classified into the ‘mismatch’ type, decoys of which have at least one mismatch to miRNA base 10 or 11.

Currently, only one endogenous decoy (AT3G09922, *IPS1*) is known in Arabidopsis [8]. IPS1 is a non-coding gene carrying a single miR399 target mimic site with a three nucleotide bulge between positions 10 and 11 relative to miRNA399. To begin evaluating how widely plants employ decoys for the regulation of miRNA-mediated target silencing and to glean any “natural guidelines” for decoy design, we initiated a systematic search and prediction of decoys in the *Arabidopsis* transcriptome set for all *Arabidopsis* miRNAs in miRBase [17]. We used our experimental results described above (and data not shown), which indicated that, in addition to the three-nucleotide bulge structure described previously [8], modified miRNA target sites with one to five nucleotide bulges between positions 10 and 11 or with one to two nucleotide mismatches at positions 10 and 11 can result in various degrees of miRNA inactivation, to set search parameters. In addition, we incorporated knowledge that a miRNA decoy can be integrated into the UTR of protein-coding transcripts. Using this set of rules (detailed in Methods), we predicted 324 putative decoy sites in 317 transcripts representing 260 loci (Table 1, Table S4), including the locus for the known decoy, IPS1. Although transcripts described as pseudogenes, transposable elements (TE) and “other RNA,” as annotated by The Arabidopsis Information Resource (TAIR), are represented, most predicted decoys are within protein-coding transcripts (Table 1). To analyze a distribution of predicted decoys along protein-coding transcripts, we assigned predicted decoy sites from protein-coding genes to 5′ UTR, ORF or 3′ UTR, normalizing the number of decoy sites to the length of the three features. Analysis indicates that decoy sites are predicted as abundantly in UTR regions as ORF regions (Fig. 7A). Currently, we have not evaluated the impact decoys could have on translation when resident within an ORF. While we identified a significant number of putative decoys, ranging in number of critical mismatches or insertions, the majority belong to a category of decoys with one or two mismatches (Fig. 7B) which, from our studies, possess a lower miRNA regulatory potential relative to the bulge category of decoys, suggesting that most predicted decoys may function for fine-tuning of miRNA-mediated silencing. One of the limitations of this computational prediction is that the current *Arabidopsis* transcriptome set used in this analysis has only a small number of annotated ncRNAs (detailed in Methods), and so the number of ncRNA transcripts that may function as decoys is very likely underestimated. Validation of predicted endogenous decoys is a next critical step in elucidating the role that miRNA decoys play in genetic regulation in plants.

**Table 1:** Distribution of predicted decoys in functional categories.

Category	Loci	Transcripts	Decoy Sites
Protein Coding	230	286	292
Short Peptide	1	1	1
Pseudogene	5	5	5
⊤Transposable Elements	22	22	23
Known Decoy (IPS1)	1	1	1
Other RNA	1	2	2
**Total**	**260**	**317**	**364**

miRNA decoy sites are predicted from *Arabidopsis* loci in different functional categories, including protein coding genes.

## Discussion

Typically, plants are genetically engineered to express single exogenous genes for traits, such as resistance to herbicides and insects [18]. A global challenge is increasing agricultural productivity for a growing population. This challenge may be met by creating more productive crops through the addition of traits, including improved tolerance to abiotic stresses such as drought, or increased yield potential. Such traits are usually controlled by a number of environmentally regulated genes, and complex trait engineering may require finely regulated or coordinately modified expression of multiple endogenous genes.

MicroRNAs have been proven to play crucial roles in plant growth, development, and adaptation to stresses through regulation of expression of multiple genes involved in these processes [3]. Thus, manipulation of endogenous miRNA activity may afford a significant opportunity to achieve the desired outcome of enhanced plant growth or response to the environment for increased crop yield. The miRNA decoy approach may provide a way for controlled manipulation of miRNA activity, thus expanding a range of methods for complex trait engineering in plants. One of the challenges in successfully utilizing this approach for crop improvement lies in our ability to achieve a desirable level of miRNA inactivation to avoid off-type effects that can compromise a positive output. Fine-tuning of activity of multiple miRNAs rather than significant inactivation of specific miRNAs is likely to be key for successful application of this approach for crop improvement. We found that in addition to stoichiometry-based control, which can be achieved by using an appropriate promoter to drive transgenic decoy expression, the degree of miRNA inactivation can also be regulated by manipulation of the miRNA decoy sequence itself, allowing a rheostat-like control of miRNA inactivation (Fig. 4A–B, Fig. 5). Multiple miRNA decoy sites can be expressed as a single expression cassette providing an opportunity for orchestrated inactivation of multiple miRNAs (Fig. 2A–B) and up-regulation of correspondent miRNA targets. These approaches can be variously combined to customize the degree of spectrum and inactivation of individual miRNAs.

While functional miRNA decoys can be expressed within non-protein-coding transcripts, we found that miRNA decoys can also sequester miRNAs when embedded in 3′UTRs of protein–coding transcripts. By inserting a spacer between the stop codon and the miRNA decoy, we could significantly increase the efficiency of miRNA inactivation. One possibility is that the progression of the translational machinery along the mRNA during protein elongation prevents a long-term association between the miRNA and the decoy site when immediately adjacent to or within the protein-coding region, as it has been shown in mammalian cells [12], thus decreasing decoy efficiency. An obvious practical application of this finding would be a multi-purpose expression cassette designed to express a protein of interest and concomitantly inactivate miRNAs. This also raises the possibility that endogenous miRNA decoy sites may exist in UTRs of protein-coding genes in plants and can be a part of the complex gene regulation network. While to our knowledge there are no described examples of natural, functional miRNA decoy sites within protein-coding transcripts of plant genes, a pilot analysis of the *Arabidopsis* transcriptome suggests the existence of protein-coding transcripts with miRNA decoy-like sites in the 5′and 3′ UTRs, as well as ORFs (Fig. 7). Some decoy sites, when placed in the 3′ UTR of a protein-coding cassette, may interfere with protein translation [11], indicating that potentially uncleavable decoy sites may have a dual function in plants, regulating protein translation *in cis* and inactivating miRNAs and regulating expression of a miRNA target *in trans*. Unlike Todesco et al. [11], we did not observe a significant change in GUS protein expression when the miRMON1 decoy was placed in the 5′UTR. This discrepancy can be explained by the difference in experimental design (reporter gene, transgenic cassette, miRNA and miRNA decoy sequence). This point would need more investigation and it remains to be determined how widespread *in cis* protein regulation by decoy-like sites is in plants.

Many complex traits associated with plant development and response to environmental cues are the result of coordinated expression of multiple genes controlled by key regulators such as transcription factors and/or miRNAs. Even subtle variations in the activity of some of these key regulators may result in a significant change in expression of downstream targets and dramatic differences in plant phenotypes and fitness. miR171 inactivation using transgenic decoys in *Arabidopsis* resulted in a range of phenotypes that are consistent with the role of miR171 in multiple developmental processes. The pale green leaf and altered flower phenotypes in response to miR171 inactivation in *Arabidopsis* have recently been reported and demonstrated [11]. In addition to leaf and flower phenotypes, we observed root and light-response phenotypes. Such pleiotropic phenotypes can be expected, as miR171 targets several SCL transcription factors [5] known to be involved in hormone and light signaling pathways [13], [14]. Interestingly, light–dependent diurnal oscillation of miR171 accumulation has been recently reported in *Arabidopsis* [19], also suggesting an involvement of miR171 in light signaling.

Over-expression of two different miR171 decoys in *Arabidopsis* resulted in a range of phenotypes. In most cases, the degree of observed phenotypes correlated with the degree of miR171 inactivation and correspondent miR171 target mRNA (SCL) up-regulation, for example, increased rosette leaf area, leaf growth angle, and leaf color. The detrimental impact on chlorophyll content and floral development from the miR171_3B decoy could be attenuated by the use of the miR171_2M decoy, which led to a more modest *SCL6-III* up-regulation (Fig. 6B–C), resulting in plants with increased leaf area but without a significant sacrifice in seed yield. Thus, subtle changes in the miR171 decoy sequence can fine-tune plant phenotypes, thereby eliminating undesirable effects in plant development while retaining desirable characteristics. The difference in efficacy of miRNA inactivation between bulge and mismatch decoys could be explained by the nature of the interaction between the miRNA decoy transcript and the miRNA-Argounaute complex. The association between the bulge decoy and the miRNA was almost perfectly complementary and at the same time, efficiently prevented Argounaute slicing activity. However, mismatch decoys typically have a less perfect complimentarity with the miRNA and are most likely less resistant to Argounaute slicing. In *Arabidopsis*, miR171 abundance was much more significantly depleted in events expressing the miR171_3B decoy than in events expressing the miR171_2M decoy (Fig. 5E). On the contrary, we did not notice any significant reduction of miRNA abundance in *N.benthamiana* leaves expressing miRNA decoys (data not shown). While a more detailed analysis needs to be done to answer the question about the difference in miRNA decoy activity, such a difference may have biological significance, allowing a wider range of regulation of miRNA inactivation. While measuring the mRNA level of miRNA targets in plants expressing engineered miRNA decoys could be an appropriate starting approach for selecting desirable transgenic events, miRNA target up-regulation is not always directly correlated with an observed phenotype (data not shown and [11]), suggesting a more complex regulation of some targets by miRNAs. Many miRNAs and their targets may have intricate expression patterns and so it is possible that some miRNAs affect target expression only in specific cell types, and only under particular conditions. For example, it has been reported that several miRNAs, including miR171, are diurnally regulated [19], thus the effect of the decoy may be obvious only at certain time points and may also depend on target protein stability. The existence of endogenous miRNA decoys can further complicate the ability to measure the effects of an engineered decoy. Some miRNAs may regulate their targets at the protein level, without a significant effect on mRNA level. Continued accumulation of knowledge with a high level of detail of miRNA-based target regulation in model plants and crops would further benefit the design and evaluation of engineered miRNA decoys.

MicroRNA decoy-mediated gene regulation approaches examined here identify a genetic toolbox with the flexibility to permit varying degrees of inactivation for one or more miRNAs in plants. In principle, the application of decoys for the modulation of endogenous miRNAs could be extended to regulate other small RNAs, both endogenous and engineered. Thus, miRNA decoys provide a unique tool, not only to study function of individual miRNAs, but also to engineer complex traits in plants.

## Materials and Methods

### Vector construction and *in planta* evaluation

Binary constructs for plant expression were made by inserting sequences downstream of the 35S promoter from cauliflower or figwort mosaic virus (sequence details in Table S1) and electroporated into *Agrobacterium tumafaciens* for subsequent plant transformation. *Arabidopsis thaliana* (L.) Heynh (ecotype Col-0) was stably transformed as previously described [20]. Transformed progeny were grown in soil (Hummert International, Earth City, MO) in chambers under 12 hour days, 150 µEi, 22°C and 70% relative humidity or on Murashige and Skoog basal salts agar plates [21]. Transient assays of constructs were performed by *Agrobacterium* infiltration of *Nicotiana benthamiana* as previously described [5]. When multiple constructs were co-expressed, each *Agrobacterium* strain was mixed in the ratio indicated in figures prior to infiltration. An empty vector was added when needed to achieve a total OD_600_ = 1 of *Agrobacterium* for each transformation. For each transient experiment, at least 6 independent infiltrations were performed and all experiments were repeated at least twice with representative data shown. Measurement of the chlorophyll content of transgenic *Arabidopsis* events relative to wild-type was performed using a non-destructive SPAD meter (Konica Minolta). Three leaves per plant per event were measured, including controls.

### Northern blot analysis of miRNA targets

RNA was extracted using TRIzol reagent (Invitrogen). Ten micrograms of total RNA was loaded into a 1.5% agarose gel containing formaldehyde, electrophoresed, and blotted [22]. ^32^P-labeled probes were prepared using the Invitrogen Radprime kit and hybridization was performed at 50°C in Sigma PerfectHyb buffer. Probe sequences are shown in Table S1. Final washes of blots were performed with 0.5× SSC, 0.1% SDS at 65°C. Blots were imaged on Kodak Biomax MS film as well as a Storm imaging system (GE Healthcare).

### Northern blot analysis of miRNAs

RNA was extracted using TRIzol reagent (Invitrogen). Seven micrograms of total RNA was resolved on a precast 15% TBE-Urea polyacrylamide gel (Invitrogen) and blotted to charged nylon membrane (Bio-Rad Transblot SD). A digoxigenin-UTP labeled T7 RNA probe complementary to At.miR171 was prepared using the DIG Northern Starter Kit (Roche) and hybridization was performed at 38°C in Sigma PerfectHyb buffer. Final washes of blots were performed with 0.5× SSC, 0.1% SDS at 50°C. Blots were imaged on blue x-ray film (Phenix). The membrane was then stripped and probed for At.miR159 in the same manner. Probe sequences are shown in Table S1.

### GFP immunodetection

Total protein was extracted from leaves as previously described [23] and western blots were performed [24]. Green fluorescent protein (GFP) was detected using the primary antibody Living Colors Full-Length A.v. Polyclonal Antibody (Clontech) at a 1∶1000 dilution and the secondary anti-rabbit IgG conjugated to horseradish peroxidase (Sigma) at a 1∶10000 dilution. ECL reagent (GE Lifesciences) was used for visualization by chemiluminescence upon exposure to Kodak Biomax MS film.

### β-glucuronidase (GUS) detection

*N. benthamiana* leaf punches were homogenized in 1× Passive Lysis Buffer (Promega) and GUS activity (uidA gene) was quantified using the 4-methylumbelliferone assay as described [25]. The GUS activity is reported as an average of seven independent replicates per inoculum mix, each replicate consisting of six tissue punches from an individual leaf.

### Quantitative RT-PCR

Expression analysis of endogenous *SCL6-III* gene was done as follows. RNA samples were treated with Turbo DNA-Free DNase (Ambion) and normalized to 5 ng/µL. Primers for qRT-PCR were selected using Applied Biosystems Primer Express version 2.0 software (Table S1). *Arabidopsis* rRNA 18S was used for normalization. qRT-PCR was carried out using the QuantiTect SYBR Green RT-PCR Kit (Qiagen) in an ABI7900HT following Qiagen manufacturer recommendations with a 60°C annealing temperature and 40 cycles. A final primer concentration of 400 nM for genes of interest and 100 nM for 18S control was used.

For *Arabidopsis* transgene expression analysis, leaf tissue was homogenized in a 1∶1 mixture of PBS, pH 7.4 (no CaCl_2_ or MgCl_2_) (Gibco) and Nucleic Acid Purification Lysis Solution (Applied Biosystems). The homogenate was then filtered through a 10 µM melt-blown polypropylene media column, and RNA subsequently bound by filtering through 0.45 µM PVDF membrane (Whatman). RNA wash buffer (Applied Biosystems) was used to rinse the membrane, and RNA was then eluted. RT-PCR and analysis was carried out using Taqman Master Mix (Applied Biosystems) following manufacturer's recommendations. Primer and probe sequences are listed in Table S1.

For expression analysis of GUS in *N. benthamiana*, RNA was extracted using the EZNA RNA Purification Kit (Omega BioTek). RNA samples were then treated with Turbo DNA-Free DNase (Ambion) and normalized to 5 ng/µL. Primers and probes for qRT-PCR were selected using Applied Biosystems Primer Express version 2.0 software (Table S1). All assays were validated using standard curve validation procedures. qRT-PCR was carried out using the TaqMan One-Step RT-PCR Master Mix Reagents Kit (Applied Biosystems) in an ABI7900HT following Applied Biosystems manufacture recommendations with a 60°C annealing temperature and 40 cycles. A final primer concentration of 300 nM and 200 nM probe for was used for each reaction. For 18S, a final primer concentration of 100 nM and 100 nM probe was used. Comparative gene expression (2-ddCt) was used for data analysis using 18S for normalization. The comparative gene expression data was log2 transformed for ANOVA and Student's T analysis.

### *SCL6-III* Quantitative RT-PCR statistical analysis

The signal data from SybrGreen was first processed with a log2-transformation, and a two-way ANOVA model was fit with Proc GLM in SAS [26], because the statistical design is a completely randomized design (CRD) with a factorial treatment structure [27]. A one-tailed t-test was applied to test the null hypothesis of equal expression between transgenic events and wild-type control against an alternative hypothesis of higher expression in transgenic events. Due to the nature of multiple comparisons from these t-tests, a Dunnett's test [26] was also used to control the experiment-wise error rate at 0.05 (unless otherwise noted). Results are summarized in Table S2. Separate statistical contrasts were also set up to investigate differences between the 2M and 3B constructs, and a two-tailed t-test was used and only raw p-values are reported (Table S3). Results are summarized in Table S3.

### GFP image quantification

GFP images from six independent replicates were captured using a Leica MZFLIII stereoscope equipped with appropriate filter (excitation filter 470/40 nm, emission filter 525/50 nm) and UV light source under consistent setting . Images were analyzed using Zeiss LSM Image Examiner. The total GFP intensity (T) for each image was calculated using the following formula:
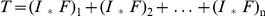
where *T* is total intensity, *I* is pixel intensity, *F* is the number of pixels at a given intensity.

### Semi-quantitative RT-PCR

RNA was extracted using TRIzol reagent (Invitrogen). RNA was normalized and treated with Turbo DNA-Free DNase (Ambion), then used for production of first strand cDNA using SuperScript III (Invitrogen). Primers specific to the miRMON1 transgene and *N. benthamiana* UBIQUITIN control (see Table S1) were designed and PCR was carried out using Phusion HS II polymerase (NEW England Biolabs) according to manufacturer's instructions. An equal volume of each PCR product was then visualized on an agarose gel.

### Computational prediction of endogenous miRNA decoys in *Arabidopsis*

For identification of miRNA decoys in *Arabidopsis*, we applied the following set of rules that has been designed based on experimental results presented in this paper and based on a set of rules developed for miRNA site prediction [28]. We required 1–5 bulges (nucleotide insertions) or nucleotide mismatches in the decoy site corresponding to miRNA bases 10–11. For mismatches to other regions of the miRNA, the following rules were applied: no bulge is allowed; a mismatch is allowed to miRNA base 1; the number of mismatches in a row should not exceed 2; there should be no more than 3 mismatches overall.

*Arabidopsis* miRNAs are from miRBase version 15, which includes 122 miRNA families and 199 members. The *Arabidopsis* cDNA set (version 9) was downloaded from TAIR (www.arabidopsis.org). In the set, there are 27,379 protein-coding sequences, 4,827 pseudogenes and transposable elements (TE), and 1,312 ‘other RNA’. To predict decoys, ‘ssearch’ from ‘fasta package’ [29] was used to thoroughly search sites in cDNA complementary to miRNAs. The search result was parsed with a Perl script which implemented the above rules.

## Supporting Information

Figure S1 **Structure of miRNA decoys and reporter cassette.** (**A**) Diagram of the interaction between bulge and mismatch miRNA decoys and a corresponding miRNA. (**B**) Diagram of the reporter gene with miRNA target site incorporated into the 3′ UTR.
(TIF)

Figure S2 **Effect of miRNA decoy structure on efficacy.** (**A**) Sequence of miRMON1 decoys with bulged (3B, 4B, 5B, 6B, 7B) structures between nucleotides 10 and 11 of the miRNA. (**B**) Expression of miRMON1-targeted GFP reporter co-expressed with 3B, 4B, 5B, 6B or 7B miRNA decoys in *N. benthamiana* leaves measured by fluorescence microscopy. Leaves were co-transformed with one part GFP reporter, 5 parts miRMON1, and 10 parts miRNA decoy. *Agrobacterium* transformed with an empty vector was added to achieve a total OD_600_ = 1 of *Agrobacterium* for each transformation.
(TIF)

Figure S3 **Leaf inclination of wild-type** ***Arabidopsis*** **and miR171 decoy events.** Rosette leaf inclination is decreased in plants expressing a miR171 decoy vs. wild-type, with the most dramatic decrease seen in miR171_3B events.
(TIF)

Figure S4 **Floral phenotypes of miR171_3B** ***Arabidopsis*** **.** miR171_3B plants exhibit a closed flower bud phenotype. Sepals and petals were removed, revealing the altered carpel.
(TIF)

Table S1 **Sequence information.**
(DOC)

Table S2 **Statistical analysis of qRT-PCR expression data of SCL6-III (At3g606030) in miR171_2B and miR171_3B plants versus wild type control.** One-sized T-test Results from ANOVA. Results are from comparison of each event (homozygous (ho) or hemizygous (he)) versus the wild type control within each tissue, and adjusted p-values were computed from Dunnett's test.
(DOC)

Table S3 **Statistical analysis of qRT-PCR expression data of SCL6-III (At3g606030) in miR171_3B transgenic decoy events versus miR171_2M transgenic decoy events.** Two-sized T-test results from ANOVA. Results are from comparison of two constructs within each tissue type. No adjustments were done to the raw p-values.
(DOC)

Table S4 **Predicted endogenous miRNA decoy sites in Arabidopsis thaliana.** For each unique miRNA sequence, the columns are miRNA ID (random if multiple loci encodes the same sequence), sequence (5′ – 3′), and all other miRNA IDs if they share the same sequence. For each predicted decoy, there are three rows listing (1) the query miRNA sequence (3′ – 5′), (2) predicted decoy site sequence and cDNA ID, decoy site coordinates of the overall cDNA length for the decoy, and (3) symbols for matches. ‘:’, match; ‘x’, canonical mismatch; ‘.’, G:U; space, indel.
(XLS)
