# Supplementary figures and images for: Regulation of Gene Expression in Plants through miRNA Inactivation
Source: PLoS One. 2011 Jun 23;6(6):e21330. doi: 10.1371/journal.pone.0021330 (PMC3121747; doi:10.1371/journal.pone.0021330)

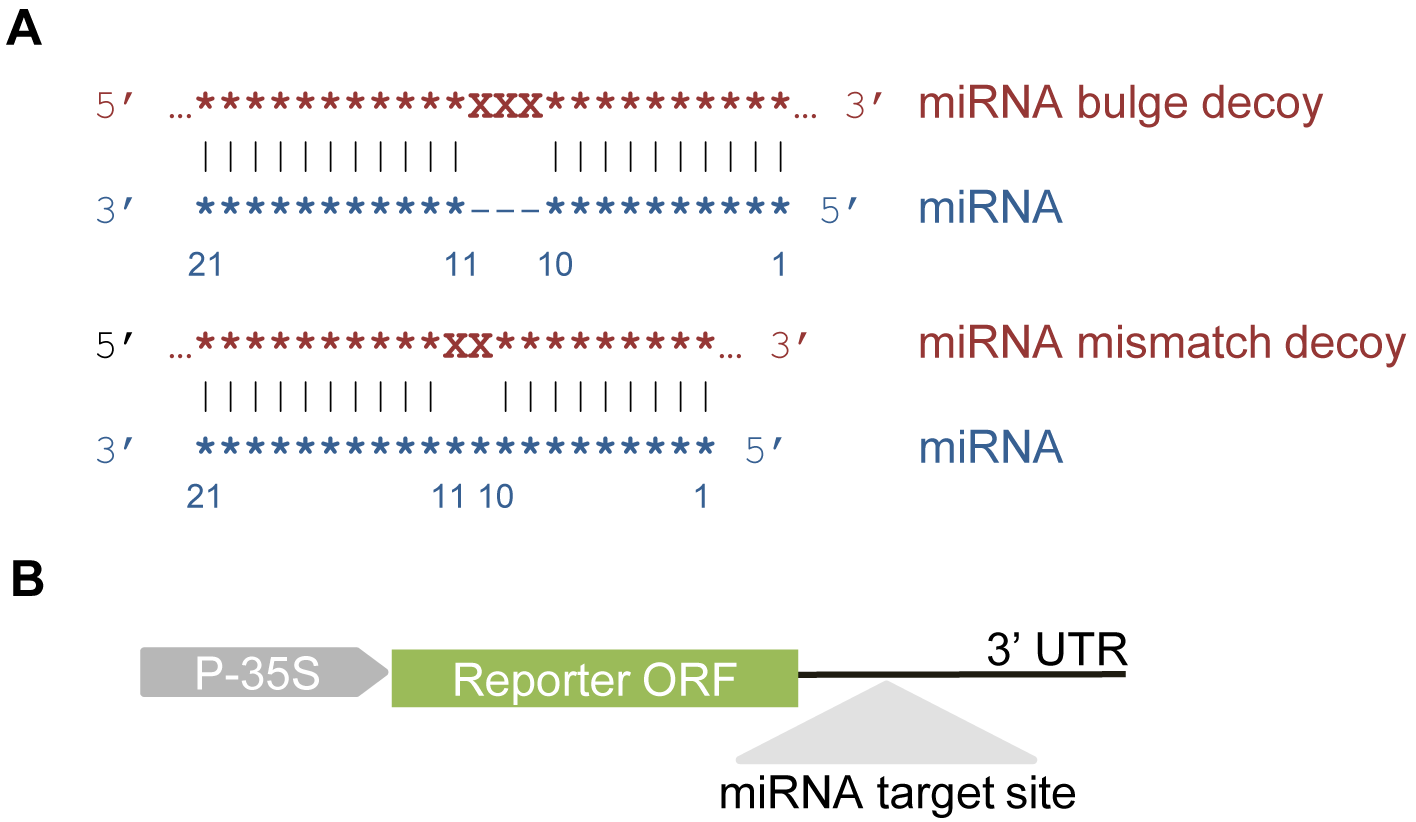

Supplement: Figure S1 — Structure of miRNA decoys and reporter cassette. (A) Diagram of the interaction between bulge and mismatch miRNA decoys and a corresponding miRNA. (B) Diagram of the reporter gene with miRNA target site incorporated into the 3′ UTR. (TIF) [file pone.0021330.s001.tif]

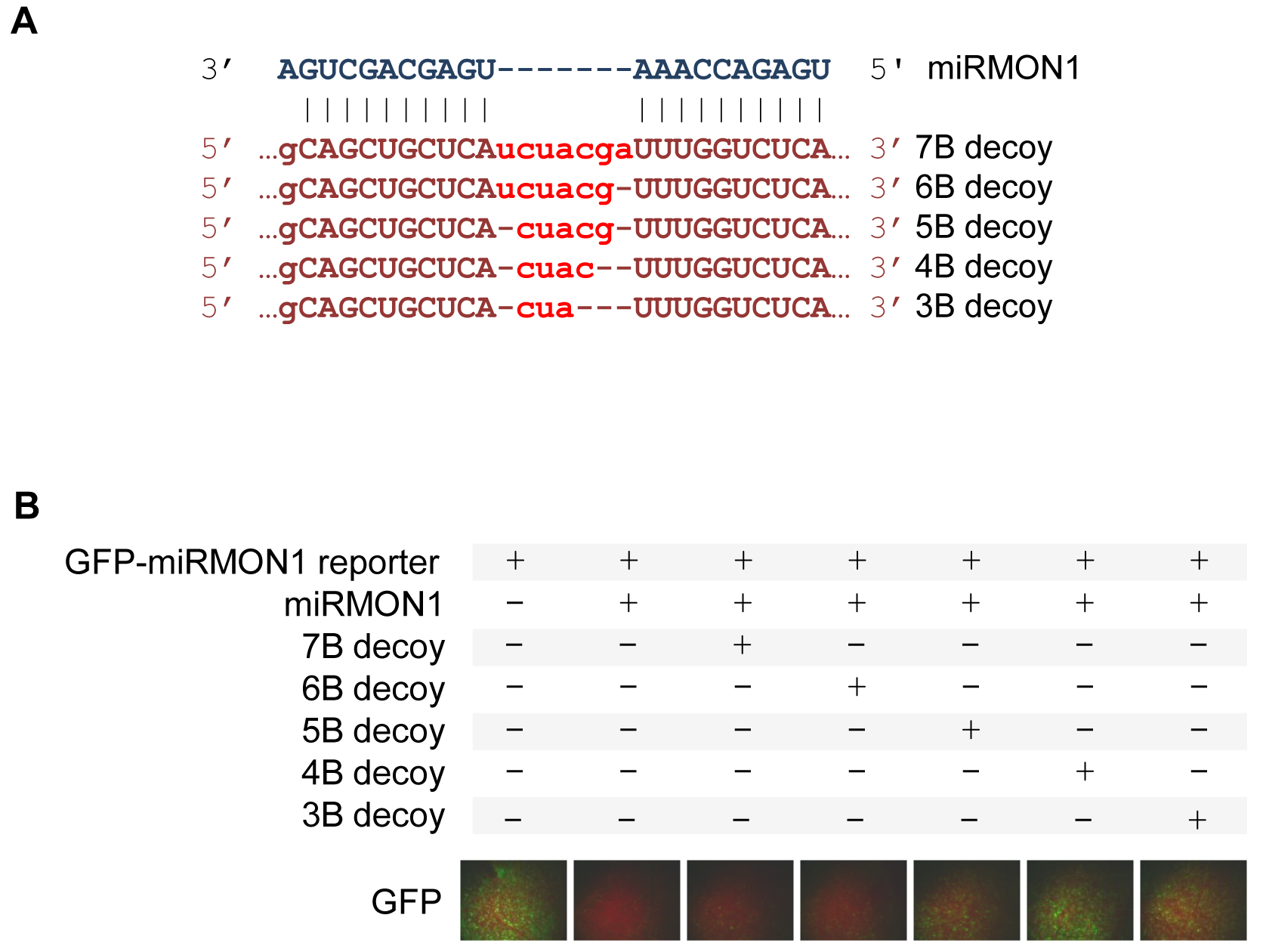

Supplement: Figure S2 — Effect of miRNA decoy structure on efficacy. (A) Sequence of miRMON1 decoys with bulged (3B, 4B, 5B, 6B, 7B) structures between nucleotides 10 and 11 of the miRNA. (B) Expression of miRMON1-targeted GFP reporter co-expressed with 3B, 4B, 5B, 6B or 7B miRNA decoys in N. benthamiana leaves measured by fluorescence microscopy. Leaves were co-transformed with one part GFP reporter, 5 parts miRMON1, and 10 parts miRNA decoy. Agrobacterium transformed with an empty vector was added to achieve a total OD600 = 1 of Agrobacterium for each transformation. (TIF) [file pone.0021330.s002.tif]

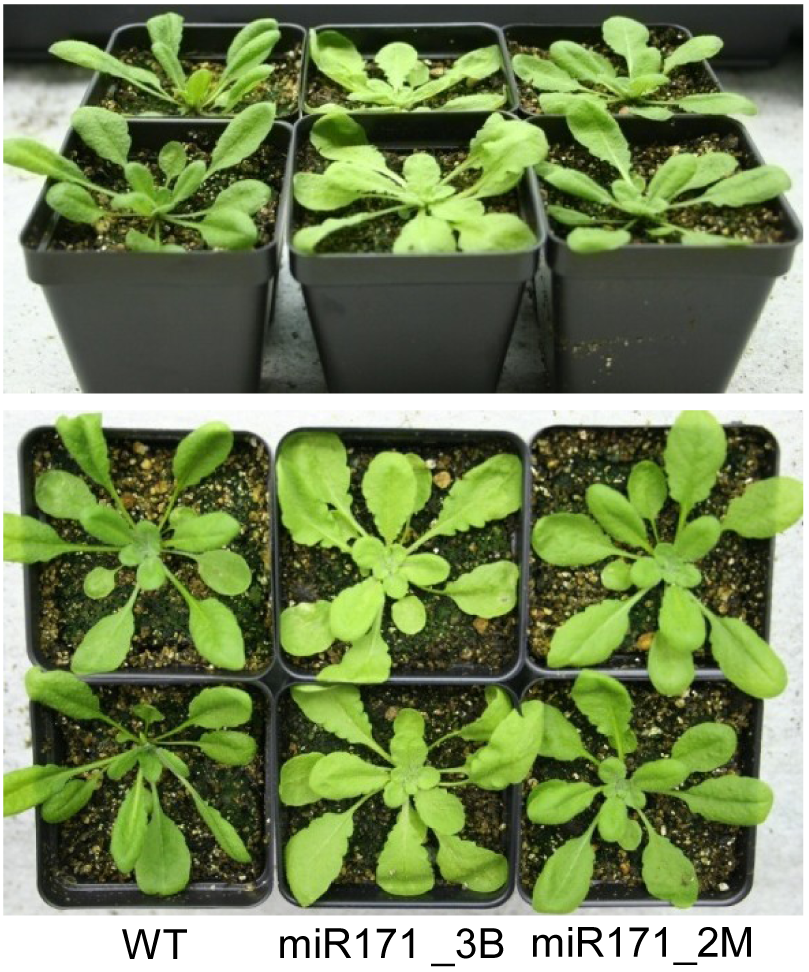

Supplement: Figure S3 — Leaf inclination of wild-type Arabidopsis and miR171 decoy events. Rosette leaf inclination is decreased in plants expressing a miR171 decoy vs. wild-type, with the most dramatic decrease seen in miR171_3B events. (TIF) [file pone.0021330.s003.tif]

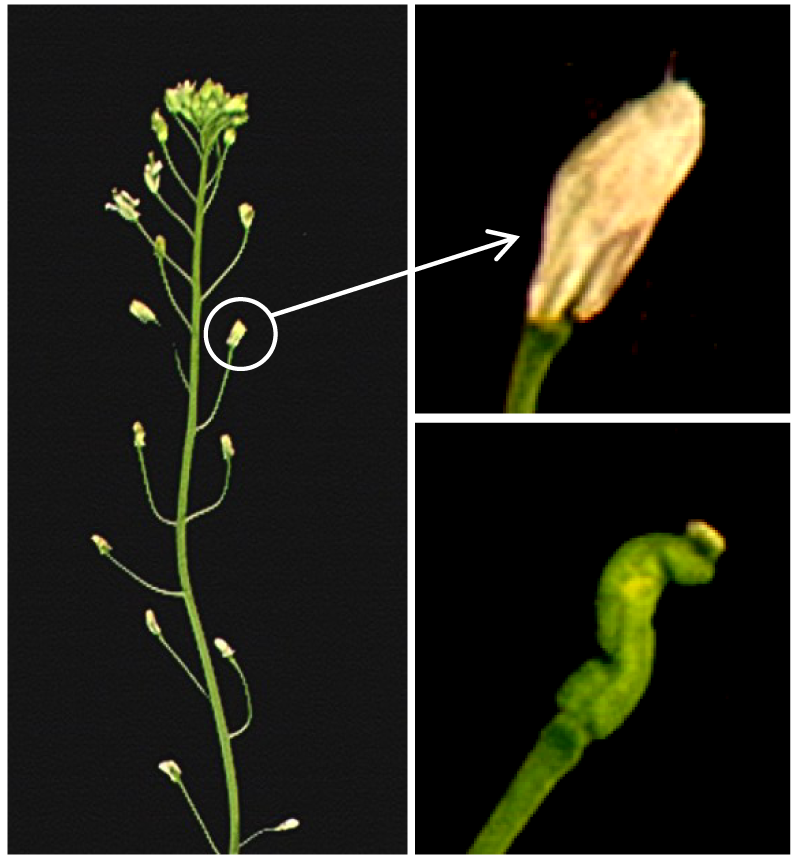

Supplement: Figure S4 — Floral phenotypes of miR171_3B Arabidopsis . miR171_3B plants exhibit a closed flower bud phenotype. Sepals and petals were removed, revealing the altered carpel. (TIF) [file pone.0021330.s004.tif]
