# Supplementary material for: Regulation of Gene Expression in Plants through miRNA Inactivation
Source: PLoS One. 2011 Jun 23;6(6):e21330. doi: 10.1371/journal.pone.0021330 (PMC3121747; doi:10.1371/journal.pone.0021330)
Supplement: Table S1 — Sequence information. (DOC) [file pone.0021330.s005.doc]

**Table S1. Sequence information**

|  | Description | Sequence |
| --- | --- | --- |
| 1 | Gma-miRMON1^1^ | 5’ UGAGACCAAAUGAGCAGCUGA 3’ |
| 2 | a-miRGL1^1^ | 5’ UGAUGAACAAUGACGGUGGAG 3’ |
| 3 | Ath-miR173^1^ | 5’ UUCGCUUGCAGAGAGAAAUCAC 3’ |
| 4 | Zma-miR528^1^ | 5’ UGGAAGGGGCAUGCAGAGGAG 3’ |
| 5 | Zma-miR399 MIM  non-coding RNA  with non-cleavable miR399 site^2^ | CCUCAAAGAGGCAGCCAUGGCUCCACAUAAAAGCGCCCAAUGGGAGCUCCCUCUUCUCCCCCCACGCCCCUCCCCCUCUUGAUAAGCCCACGUCGGCGGCAGGGGGGGCGGCCGCCAGGCUUGCUAUAGCUGGUCCAUGGCACCAUACAUGUAAGCACGCACACAGGCACACACACACACGCACGCAAUGAUCUACGUAUCUAGCAGCAGCUUAUCAUGUCGUCAUCAUGCAUGCAUGGCCGACGGAGGUCGUCAUCUUAUCUGGGAGCGUGUGUGUCUUGGCAAUGGGAAGCUGCAUGCGCCUCUCGGGCGUCGGCGCGUCGGCGCCUAGCUGUAGGGCGGCGUGCCAUAGAGCUGCCUCCUGCCGCUCACACCAUGCUGUUGACGAGGACUGAUGGUGGCCAUGGCCUCUCGGCGUCGGUGGCGGCGGCGCCGGCGCCGAGUUUUACCUCUCUACUAAGG**UAGGGCAACUUGUAUCCUUUGGCA**AUUGUUCUCAUCUAUCUGGGUCUGUCUGUUGGCUGCCCGGUGACGGUAUACGGUGAUGUUCUAAUAGUACUCAAUUGGUCUUGGAUCGGAGUUCAUGCUACGGCUCCUCUGUUAUAUAUUACACGGCUGACGGCUCCUCCUUAUUAAUGUGUAC |
| 6 | miR399 MIM/miRMON1 Decoy Site^3,4^ | GCAGCUGCUCACUAUUUGGUCUCA |
| 7 | Gma-miRMON1  precursor^4,5^ | AAUUCAUUACAUUGAUAAAACACAAUUCAAAAGAUCAAUGUUCCACUUCAUGCAAAGACAUUUCCAAAAUAUGUGUAGGUAGAGGGGUUUUACAGGAUCGUCC**UGAGACCAAAUGAGCAGCUGA**CCACAUGAUGCAGCUAUGUUUGCUAU**UCAGCUGCUCAUCUGUUCUCA**GGUCGCCCUUGUUGGACUGUCCAACUCCUACUGAUUGCGGAUGCACUUGCCACAAAUGAAAAUCAAAGCGAGGGGAAAAGAAUGUAGAGUGUGACUACGAUUGCAUGCAUGUGAUUUAGGUAAUUAAGUUACAUGAUUGUCUAAUUGUGUUUAUGGAAUUGUAUA |
| 8 | GUS/miRMON1 Decoy^2,4,6^ | AUGGUCCGUCCUGUAGAAACCCCAACCCGUGAAAUCAAAAAACUCGACGGCCUGUGGGCAUUCAGUCUGGAUCGCGAAAACUGUGGAAUUGAUCAGCGUUGGUGGGAAAGCGCGUUACAAGAAAGCCGGGCAAUUGCUGUGCCAGGCAGUUUUAACGAUCAGUUCGCCGAUGCAGAUAUUCGUAAUUAUGCGGGCAACGUCUGGUAUCAGCGCGAAGUCUUUAUACCGAAAGGUUGGGCAGGCCAGCGUAUCGUGCUGCGUUUCGAUGCGGUCACUCAUUACGGCAAAGUGUGGGUCAAUAAUCAGGAAGUGAUGGAGCAUCAGGGCGGCUAUACGCCAUUUGAAGCCGAUGUCACGCCGUAUGUUAUUGCCGGGAAAAGUGUACGUAAGUUUCUGCUUCUACCUUUGAUAUAUAUAUAAUAAUUAUCAUUAAUUAGUAGUAAUAUAAUAUUUCAAAUAUUUUUUUCAAAAUAAAAGAAUGUAGUAUAUAGCAAUUGCUUUUCUGUAGUUUAUAAGUGUGUAUAUUUUAAUUUAUAACUUUUCUAAUAUAUGACCAAAAUUUGUUGAUGUGCAGGUAUCACCGUUUGUGUGAACAACGAACUGAACUGGCAGACUAUCCCGCCGGGAAUGGUGAUUACCGACGAAAACGGCAAGAAAAAGCAGUCUUACUUCCAUGAUUUCUUUAACUAUGCCGGAAUCCAUCGCAGCGUAAUGCUCUACACCACGCCGAACACCUGGGUGGACGAUAUCACCGUGGUGACGCAUGUCGCGCAAGACUGUAACCACGCGUCUGUUGACUGGCAGGUGGUGGCCAAUGGUGAUGUCAGCGUUGAACUGCGUGAUGCGGAUCAACAGGUGGUUGCAACUGGACAAGGCACUAGCGGGACUUUGCAAGUGGUGAAUCCGCACCUCUGGCAACCGGGUGAAGGUUAUCUCUAUGAACUGUGCGUCACAGCCAAAAGCCAGACAGAGUGUGAUAUCUACCCGCUUCGCGUCGGCAUCCGGUCAGUGGCAGUGAAGGGCGAACAGUUCCUGAUUAACCACAAACCGUUCUACUUUACUGGCUUUGGUCGUCAUGAAGAUGCGGACUUGCGUGGCAAAGGAUUCGAUAACGUGCUGAUGGUGCACGACCACGCAUUAAUGGACUGGAUUGGGGCCAACUCCUACCGUACCUCGCAUUACCCUUACGCUGAAGAGAUGCUCGACUGGGCAGAUGAACAUGGCAUCGUGGUGAUUGAUGAAACUGCUGCUGUCGGCUUUAACCUCUCUUUAGGCAUUGGUUUCGAAGCGGGCAACAAGCCGAAAGAACUGUACAGCGAAGAGGCAGUCAACGGGGAAACUCAGCAAGCGCACUUACAGGCGAUUAAAGAGCUGAUAGCGCGUGACAAAAACCACCCAAGCGUGGUGAUGUGGAGUAUUGCCAACGAACCGGAUACCCGUCCGCAAGGUGCACGGGAAUAUUUCGCGCCACUGGCGGAAGCAACGCGUAAACUCGACCCGACGCGUCCGAUCACCUGCGUCAAUGUAAUGUUCUGCGACGCUCACACCGAUACCAUCAGCGAUCUCUUUGAUGUGCUGUGCCUGAACCGUUAUUACGGAUGGUAUGUCCAAAGCGGCGAUUUGGAAACGGCAGAGAAGGUACUGGAAAAAGAACUUCUGGCCUGGCAGGAGAAACUGCAUCAGCCGAUUAUCAUCACCGAAUACGGCGUGGAUACGUUAGCCGGGCUGCACUCAAUGUACACCGACAUGUGGAGUGAAGAGUAUCAGUGUGCAUGGCUGGAUAUGUAUCACCGCGUCUUUGAUCGCGUCAGCGCCGUCGUCGGUGAACAGGUAUGGAAUUUCGCCGAUUUUGCGACCUCGCAAGGCAUAUUGCGCGUUGGCGGUAACAAGAAAGGGAUCUUCACUCGCGACCGCAAACCGAAGUCGGCGGCUUUUCUGCUGCAAAAACGCUGGACUGGCAUGAACUUCGGUGAAAAACCGCAGCAGGGAGGCAAACAAUGA**GCAGCUGCUCACUAUUUGGUCUCA** |
| 9 | GUS+Spacer/miRMON1 Decoy^2,4,6,7^ | AUGGUCCGUCCUGUAGAAACCCCAACCCGUGAAAUCAAAAAACUCGACGGCCUGUGGGCAUUCAGUCUGGAUCGCGAAAACUGUGGAAUUGAUCAGCGUUGGUGGGAAAGCGCGUUACAAGAAAGCCGGGCAAUUGCUGUGCCAGGCAGUUUUAACGAUCAGUUCGCCGAUGCAGAUAUUCGUAAUUAUGCGGGCAACGUCUGGUAUCAGCGCGAAGUCUUUAUACCGAAAGGUUGGGCAGGCCAGCGUAUCGUGCUGCGUUUCGAUGCGGUCACUCAUUACGGCAAAGUGUGGGUCAAUAAUCAGGAAGUGAUGGAGCAUCAGGGCGGCUAUACGCCAUUUGAAGCCGAUGUCACGCCGUAUGUUAUUGCCGGGAAAAGUGUACGUAAGUUUCUGCUUCUACCUUUGAUAUAUAUAUAAUAAUUAUCAUUAAUUAGUAGUAAUAUAAUAUUUCAAAUAUUUUUUUCAAAAUAAAAGAAUGUAGUAUAUAGCAAUUGCUUUUCUGUAGUUUAUAAGUGUGUAUAUUUUAAUUUAUAACUUUUCUAAUAUAUGACCAAAAUUUGUUGAUGUGCAGGUAUCACCGUUUGUGUGAACAACGAACUGAACUGGCAGACUAUCCCGCCGGGAAUGGUGAUUACCGACGAAAACGGCAAGAAAAAGCAGUCUUACUUCCAUGAUUUCUUUAACUAUGCCGGAAUCCAUCGCAGCGUAAUGCUCUACACCACGCCGAACACCUGGGUGGACGAUAUCACCGUGGUGACGCAUGUCGCGCAAGACUGUAACCACGCGUCUGUUGACUGGCAGGUGGUGGCCAAUGGUGAUGUCAGCGUUGAACUGCGUGAUGCGGAUCAACAGGUGGUUGCAACUGGACAAGGCACUAGCGGGACUUUGCAAGUGGUGAAUCCGCACCUCUGGCAACCGGGUGAAGGUUAUCUCUAUGAACUGUGCGUCACAGCCAAAAGCCAGACAGAGUGUGAUAUCUACCCGCUUCGCGUCGGCAUCCGGUCAGUGGCAGUGAAGGGCGAACAGUUCCUGAUUAACCACAAACCGUUCUACUUUACUGGCUUUGGUCGUCAUGAAGAUGCGGACUUGCGUGGCAAAGGAUUCGAUAACGUGCUGAUGGUGCACGACCACGCAUUAAUGGACUGGAUUGGGGCCAACUCCUACCGUACCUCGCAUUACCCUUACGCUGAAGAGAUGCUCGACUGGGCAGAUGAACAUGGCAUCGUGGUGAUUGAUGAAACUGCUGCUGUCGGCUUUAACCUCUCUUUAGGCAUUGGUUUCGAAGCGGGCAACAAGCCGAAAGAACUGUACAGCGAAGAGGCAGUCAACGGGGAAACUCAGCAAGCGCACUUACAGGCGAUUAAAGAGCUGAUAGCGCGUGACAAAAACCACCCAAGCGUGGUGAUGUGGAGUAUUGCCAACGAACCGGAUACCCGUCCGCAAGGUGCACGGGAAUAUUUCGCGCCACUGGCGGAAGCAACGCGUAAACUCGACCCGACGCGUCCGAUCACCUGCGUCAAUGUAAUGUUCUGCGACGCUCACACCGAUACCAUCAGCGAUCUCUUUGAUGUGCUGUGCCUGAACCGUUAUUACGGAUGGUAUGUCCAAAGCGGCGAUUUGGAAACGGCAGAGAAGGUACUGGAAAAAGAACUUCUGGCCUGGCAGGAGAAACUGCAUCAGCCGAUUAUCAUCACCGAAUACGGCGUGGAUACGUUAGCCGGGCUGCACUCAAUGUACACCGACAUGUGGAGUGAAGAGUAUCAGUGUGCAUGGCUGGAUAUGUAUCACCGCGUCUUUGAUCGCGUCAGCGCCGUCGUCGGUGAACAGGUAUGGAAUUUCGCCGAUUUUGCGACCUCGCAAGGCAUAUUGCGCGUUGGCGGUAACAAGAAAGGGAUCUUCACUCGCGACCGCAAACCGAAGUCGGCGGCUUUUCUGCUGCAAAAACGCUGGACUGGCAUGAACUUCGGUGAAAAACCGCAGCAGGGAGGCAAACAAUGAAAGUACUCCGAUCGCGUUAACGCUUUAUCACGAUACCUUCUACCACAUAUCACUAACAACAUCAACACUCAUCAC**GCAGCUGCUCACUAUUUGGUCUCA** |
| 10 | miRMON1/miRMON1 Decoy^2,8^ | AAUUCAUUACAUUGAUAAAACACAAUUCAAAAGAUCAAUGUUCCACUUCAUGCAAAGACAUUUCCAAAAUAUGUGUAGGUAGAGGGGUUUUACAGGAUCGUCC**GCAGCUGCUCACUAUUUGGUCUCA**GGUCGCCCUUGUUGGACUGUCCAACUCCUACUGAUUGCGGAUGCACUUGCCACAAAUGAAAAUCAAAGCGAGGGGAAAAGAAUGUAGAGUGUGACUACGAUUGCAUGCAUGUGAUUUAGGUAAUUAAGUUACAUGAUUGUCUAAUUGUGUUUAUGGAAUUGUAUA |
| 11 | GFP/miRMON1 Reporter^4,6,9^ | AUGGGCAAGGGCGAGGAACUGUUCACUGGCGUGGUCCCAAUCCUGGUGGAACUGGAUGGUGAUGUGAACGGGCACAAGUUCUCCGUCAGCGGAGAGGGUGAAGGUGAUGCCACCUACGGAAAGCUCACCCUGAAGUUCAUCUGCACUACCGGAAAGCUCCCUGUUCCGUGGCCAACCCUCGUCACCACUUUCACCUACGGUGUUCAGUGCUUCUCCCGGUACCCAGAUCACAUGAAGCAGCAUGACUUCUUCAAGAGCGCCAUGCCCGAAGGCUACGUGCAAGAAAGGACUAUCUUCUUCAAGGAUGACGGGAACUACAAGACACGUGCCGAAGUCAAGUUCGAAGGUGAUACCCUGGUGAACCGCAUCGAGCUGAAAGGUAAGUUUCUGCUUCUACCUUUGAUAUAUAUAUAAUAAUUAUCAUUAAUUAGUAGUAAUAUAAUAUUUCAAAUAUUUUUUUCAAAAUAAAAGAAUGUAGUAUAUAGCAAUUGCUUUUCUGUAGUUUAUAAGUGUGUAUAUUUUAAUUUAUAACUUUUCUAAUAUAUGACCAAAAUUUGUUGAUGUGCAGGUAUCGAUUUCAAGGAAGAUGGAAACAUCCUCGGACACAAGCUGGAGUACAACUACAACUCCCACAACGUAUACAUCAUGGCCGACAAGCAGAAGAACGGCAUCAAGGUGAACUUCAAGAUCAGGCACAACAUCGAAGAUGGAAGCGUGCAACUGGCGGACCACUACCAGCAGAACACGCCCAUCGGCGAUGGCCCUGUCCUGCUGCCGGACAACCAUUACCUGUCCACGCAAUCUGCCCUCUCCAAGGACCCCAACGAGAAGAGGGACCACAUGGUCCUGCUGGAGUUCGUGACGGCUGCUGGGAUCACGCAUGGCAUGGAUGAACUCUACAAGUGAAGAUC**UCCAGCUGCUCAUUUGGUCUCA**UGAUGCCAUAGAUCACUUGAUGUCACGACCACCGUCAUUGUUCAUCAGAUUUCUCUCUGCAAGCGAA |
| 12 | GUS+Spacer/Decoy^6,7,11^ | AUGGUCCGUCCUGUAGAAACCCCAACCCGUGAAAUCAAAAAACUCGACGGCCUGUGGGCAUUCAGUCUGGAUCGCGAAAACUGUGGAAUUGAUCAGCGUUGGUGGGAAAGCGCGUUACAAGAAAGCCGGGCAAUUGCUGUGCCAGGCAGUUUUAACGAUCAGUUCGCCGAUGCAGAUAUUCGUAAUUAUGCGGGCAACGUCUGGUAUCAGCGCGAAGUCUUUAUACCGAAAGGUUGGGCAGGCCAGCGUAUCGUGCUGCGUUUCGAUGCGGUCACUCAUUACGGCAAAGUGUGGGUCAAUAAUCAGGAAGUGAUGGAGCAUCAGGGCGGCUAUACGCCAUUUGAAGCCGAUGUCACGCCGUAUGUUAUUGCCGGGAAAAGUGUACGUAAGUUUCUGCUUCUACCUUUGAUAUAUAUAUAAUAAUUAUCAUUAAUUAGUAGUAAUAUAAUAUUUCAAAUAUUUUUUUCAAAAUAAAAGAAUGUAGUAUAUAGCAAUUGCUUUUCUGUAGUUUAUAAGUGUGUAUAUUUUAAUUUAUAACUUUUCUAAUAUAUGACCAAAAUUUGUUGAUGUGCAGGUAUCACCGUUUGUGUGAACAACGAACUGAACUGGCAGACUAUCCCGCCGGGAAUGGUGAUUACCGACGAAAACGGCAAGAAAAAGCAGUCUUACUUCCAUGAUUUCUUUAACUAUGCCGGAAUCCAUCGCAGCGUAAUGCUCUACACCACGCCGAACACCUGGGUGGACGAUAUCACCGUGGUGACGCAUGUCGCGCAAGACUGUAACCACGCGUCUGUUGACUGGCAGGUGGUGGCCAAUGGUGAUGUCAGCGUUGAACUGCGUGAUGCGGAUCAACAGGUGGUUGCAACUGGACAAGGCACUAGCGGGACUUUGCAAGUGGUGAAUCCGCACCUCUGGCAACCGGGUGAAGGUUAUCUCUAUGAACUGUGCGUCACAGCCAAAAGCCAGACAGAGUGUGAUAUCUACCCGCUUCGCGUCGGCAUCCGGUCAGUGGCAGUGAAGGGCGAACAGUUCCUGAUUAACCACAAACCGUUCUACUUUACUGGCUUUGGUCGUCAUGAAGAUGCGGACUUGCGUGGCAAAGGAUUCGAUAACGUGCUGAUGGUGCACGACCACGCAUUAAUGGACUGGAUUGGGGCCAACUCCUACCGUACCUCGCAUUACCCUUACGCUGAAGAGAUGCUCGACUGGGCAGAUGAACAUGGCAUCGUGGUGAUUGAUGAAACUGCUGCUGUCGGCUUUAACCUCUCUUUAGGCAUUGGUUUCGAAGCGGGCAACAAGCCGAAAGAACUGUACAGCGAAGAGGCAGUCAACGGGGAAACUCAGCAAGCGCACUUACAGGCGAUUAAAGAGCUGAUAGCGCGUGACAAAAACCACCCAAGCGUGGUGAUGUGGAGUAUUGCCAACGAACCGGAUACCCGUCCGCAAGGUGCACGGGAAUAUUUCGCGCCACUGGCGGAAGCAACGCGUAAACUCGACCCGACGCGUCCGAUCACCUGCGUCAAUGUAAUGUUCUGCGACGCUCACACCGAUACCAUCAGCGAUCUCUUUGAUGUGCUGUGCCUGAACCGUUAUUACGGAUGGUAUGUCCAAAGCGGCGAUUUGGAAACGGCAGAGAAGGUACUGGAAAAAGAACUUCUGGCCUGGCAGGAGAAACUGCAUCAGCCGAUUAUCAUCACCGAAUACGGCGUGGAUACGUUAGCCGGGCUGCACUCAAUGUACACCGACAUGUGGAGUGAAGAGUAUCAGUGUGCAUGGCUGGAUAUGUAUCACCGCGUCUUUGAUCGCGUCAGCGCCGUCGUCGGUGAACAGGUAUGGAAUUUCGCCGAUUUUGCGACCUCGCAAGGCAUAUUGCGCGUUGGCGGUAACAAGAAAGGGAUCUUCACUCGCGACCGCAAACCGAAGUCGGCGGCUUUUCUGCUGCAAAAACGCUGGACUGGCAUGAACUUCGGUGAAAAACCGCAGCAGGGAGGCAAACAAUGAAAGUACUCCGAUCGCGUUAACGCUUUAUCACGAUACCUUCUACCACAUAUCACUAACAACAUCAACACUCAUCAC-(**Decoy Site)** |
| 13 | miRMON1_1B^4,10^ | GCAGCUGCUCACUUUGGUCUCA |
| 14 | miRMON1_2B^4,10^ | GCAGCUGCUCAUAUUUGGUCUCA |
| 15 | miRMON1_3B^4,10^ | GCAGCUGCUCACUAUUUGGUCUCA |
| 16 | miRMON1_4B^4,10^ | GCAGCUGCUCACUACUUUGGUCUCA |
| 17 | miRMON1_5B^4,10^ | GCAGCUGCUCAcuacgUUUGGUCUCA |
| 18 | miRMON1_6B^4,10^ | GCAGCUGCUCAUcuacgUUUGGUCUCA |
| 19 | miRMON1_7B^4,10^ | GCAGCUGCUCAUcuacgAUUUGGUCUCA |
| 20 | miRMON1_1M (10)^4,10^ | GCAGCUGCUCACUUGGUCUCA |
| 21 | miRMON1_1M (11)^4,10^ | GCAGCUGCUCUUUUGGUCUCA |
| 22 | miRMON1_1M (12)^4,10^ | GCAGCUGCUGAUUUGGUCUCA |
| 23 | miRMON1_2M (10,11)^4,10^ | GCAGCUGCUCUCUUGGUCUCA |
| 24 | miRMON1_2M (11,12)^4,10^ | GCAGCUGCUGUUUUGGUCUCA |
| 25 | miRMON1_ 3M (10,11,12)^4,10^ | GCAGCUGCUGUCUUGGUCUCA |
| 26 | GUS-miRGL1 Reporter^6,9,12^ | AUGGUCCGUCCUGUAGAAACCCCAACCCGUGAAAUCAAAAAACUCGACGGCCUGUGGGCAUUCAGUCUGGAUCGCGAAAACUGUGGAAUUGAUCAGCGUUGGUGGGAAAGCGCGUUACAAGAAAGCCGGGCAAUUGCUGUGCCAGGCAGUUUUAACGAUCAGUUCGCCGAUGCAGAUAUUCGUAAUUAUGCGGGCAACGUCUGGUAUCAGCGCGAAGUCUUUAUACCGAAAGGUUGGGCAGGCCAGCGUAUCGUGCUGCGUUUCGAUGCGGUCACUCAUUACGGCAAAGUGUGGGUCAAUAAUCAGGAAGUGAUGGAGCAUCAGGGCGGCUAUACGCCAUUUGAAGCCGAUGUCACGCCGUAUGUUAUUGCCGGGAAAAGUGUACGUAAGUUUCUGCUUCUACCUUUGAUAUAUAUAUAAUAAUUAUCAUUAAUUAGUAGUAAUAUAAUAUUUCAAAUAUUUUUUUCAAAAUAAAAGAAUGUAGUAUAUAGCAAUUGCUUUUCUGUAGUUUAUAAGUGUGUAUAUUUUAAUUUAUAACUUUUCUAAUAUAUGACCAAAAUUUGUUGAUGUGCAGGUAUCACCGUUUGUGUGAACAACGAACUGAACUGGCAGACUAUCCCGCCGGGAAUGGUGAUUACCGACGAAAACGGCAAGAAAAAGCAGUCUUACUUCCAUGAUUUCUUUAACUAUGCCGGAAUCCAUCGCAGCGUAAUGCUCUACACCACGCCGAACACCUGGGUGGACGAUAUCACCGUGGUGACGCAUGUCGCGCAAGACUGUAACCACGCGUCUGUUGACUGGCAGGUGGUGGCCAAUGGUGAUGUCAGCGUUGAACUGCGUGAUGCGGAUCAACAGGUGGUUGCAACUGGACAAGGCACUAGCGGGACUUUGCAAGUGGUGAAUCCGCACCUCUGGCAACCGGGUGAAGGUUAUCUCUAUGAACUGUGCGUCACAGCCAAAAGCCAGACAGAGUGUGAUAUCUACCCGCUUCGCGUCGGCAUCCGGUCAGUGGCAGUGAAGGGCGAACAGUUCCUGAUUAACCACAAACCGUUCUACUUUACUGGCUUUGGUCGUCAUGAAGAUGCGGACUUGCGUGGCAAAGGAUUCGAUAACGUGCUGAUGGUGCACGACCACGCAUUAAUGGACUGGAUUGGGGCCAACUCCUACCGUACCUCGCAUUACCCUUACGCUGAAGAGAUGCUCGACUGGGCAGAUGAACAUGGCAUCGUGGUGAUUGAUGAAACUGCUGCUGUCGGCUUUAACCUCUCUUUAGGCAUUGGUUUCGAAGCGGGCAACAAGCCGAAAGAACUGUACAGCGAAGAGGCAGUCAACGGGGAAACUCAGCAAGCGCACUUACAGGCGAUUAAAGAGCUGAUAGCGCGUGACAAAAACCACCCAAGCGUGGUGAUGUGGAGUAUUGCCAACGAACCGGAUACCCGUCCGCAAGGUGCACGGGAAUAUUUCGCGCCACUGGCGGAAGCAACGCGUAAACUCGACCCGACGCGUCCGAUCACCUGCGUCAAUGUAAUGUUCUGCGACGCUCACACCGAUACCAUCAGCGAUCUCUUUGAUGUGCUGUGCCUGAACCGUUAUUACGGAUGGUAUGUCCAAAGCGGCGAUUUGGAAACGGCAGAGAAGGUACUGGAAAAAGAACUUCUGGCCUGGCAGGAGAAACUGCAUCAGCCGAUUAUCAUCACCGAAUACGGCGUGGAUACGUUAGCCGGGCUGCACUCAAUGUACACCGACAUGUGGAGUGAAGAGUAUCAGUGUGCAUGGCUGGAUAUGUAUCACCGCGUCUUUGAUCGCGUCAGCGCCGUCGUCGGUGAACAGGUAUGGAAUUUCGCCGAUUUUGCGACCUCGCAAGGCAUAUUGCGCGUUGGCGGUAACAAGAAAGGGAUCUUCACUCGCGACCGCAAACCGAAGUCGGCGGCUUUUCUGCUGCAAAAACGCUGGACUGGCAUGAACUUCGGUGAAAAACCGCAGCAGGGAGGCAAACAAUGAGGAUCCCGAUCGAUUCUU**GACCACCGUCAUUGUUCA**UCAAAGCUU |
| 27 | a-miRGL1^5,8^ | AAUUCAUUACAUUGAUAAAACACAAUUCAAAAGAUCAAUGUUCCACUUCAUGCAAAGACAUUUCCAAAAUAUGUGUAGGUAGAGGGGUUUUACAGGAUCGUCC**UGAUGAACAAUGACGGUGGAG**CCACAUGAUGCAGCUAUGUUUGCUAU**CUCCACCGUCAUCGUCCAUCA**GGUCGCCCUUGUUGGACUGUCCAACUCCUACUGAUUGCGGAUGCACUUGCCACAAAUGAAAAUCAAAGCGAGGGGAAAAGAAUGUAGAGUGUGACUACGAUUGCAUGCAUGUGAUUUAGGUAAUUAAGUUACAUGAUUGUCUAAUUGUGUUUAUGGAAUUGUAUA |
| 28 | miRGL1-miRMON1 Double Decoy^2,3,4,7,12^ | CCUCAAAGAGGCAGCCAUGGCUCCACAUAAAAGCGCCCAAUGGGAGCUCCCUCUUCUCCCCCCACGCCCCUCCCCCUCUUGAUAAGCCCACGUCGGCGGCAGGGGGGGCGGCCGCCAGGCUUGCUAUAGCUGGUCCAUGGCACCAUACAUGUAAGCACGCACACAGGCACACACACACACGCACGCAAUGAUCUACGUAUCUAGCAGCAGCUUAUCAUGUCGUCAUCAUGCAUGCAUGGCCGACGGAGGUCGUCAUCUUAUCUGGGAGCGUGUGUGUCUUGGCAAUGGGAAGCUGCAUGCGCCUCUCGGGCGUCGGCGCGUCGGCGCCUAGCUGUAGGGCGGCGUGCCAUAGAGCUGCCUCCUGCCGCUCACACCAUGCUGUUGACGAGGACUGAUGGUGGCCAUGGCCUCUCGGCGUCGGUGGCGGCGGCGCCGGCGCCGAGUUUUACCUCUCUACUAAGG**GUCCACCGUCACUGUUGUUCAUCA**AAGUACUCCGAUCGCGUUAACGCUUUAUCACGAUACCUUCUACCACAUAUCACUAACAACAUCAACACUCAUCAC**GCAGCUGCUCACUAUUUGGUCUCA**AUUGUUCUCAUCUAUCUGGGUCUGUCUGUUGGCUGCCCGGUGACGGUAUACGGUGAUGUUCUAAUAGUACUCAAUUGGUCUUGGAUCGGAGUUCAUGCUACGGCUCCUCUGUUAUAUAUUACACGGCUGACGGCUCCUCCUUAUUAAUGUGUAC |
| 29 | miR171_3B^3,13^ | GAUAUUGGCGCAUAGGCUCAAUCA |
| 30 | miR171_2M(10,11)^3,13^ | GAUAUUGGCGUCGCUCAAUCA |
| 31 | Taqman transgene expression primer and probe sequences (GUS) – *N. benthamiana* | Forward 5’ – ACCGAATACGGCGTCCATAC – 3’  Reverse 5’ – TCCAGCCATGCACACTGATAC – 3’  FAM Probe 6FAM-TGTACACCGACATGTGG-MGB |
| 32 | Taqman expression primer and probe sequences (18S control) – *N. benthamiana* | Forward 5’ – CGTCCCTGCCCTTTGTACAC – 3’  Reverse 5’ – CGAACACTTCACCGGATCATT – 3’  VIC Probe VIC-CCGCCCGTCGCTCCTACCGAT-TAMRA |
|  |  |  |
| 33 | sqRT-PCR primers (*UBIQUITIN*) – *N. benthamiana* | Forward 5’ – ATGCAGATCTTCGTGAAGACATTG – 3’  Reverse 5’ – GAAACCACCACGGAGACGGAG – 3’ |
| 34 | sqRT-PCR transgene primers – miRMON1 | Forward 5’ – ACATGATGCAGCTATGTTTGC – 3’  Reverse 5’ – ACTGTACGACAGGTGATCAGG – 3’ |
| 35 | At SCL6-III (At3g60630) northern probe sequence | ATGCCCCTGCCCTTTGAGCAATTTCAAGGGAAGGGGGTTCTGGGTTTCTTAGATTCTTCTTCTTCTCCGGGATACAAAATCTGGGCTAATCCAGAGAAGCTCCATGGACGAGTAGAAGAAGATCTCTGCTTTGTTGTCAACAATGGTGGTTTCTCGGAGCCGACGTCTGTTTTAGACTCTGTTAGAAGTCCAAGCCCTTTCGTCTCTTCTTCAACCACCACGCTGTCTTCTTCTCACGGTGGTCCCAGCGGCGGCGGCGCTGCTGCTGCTACTTTTTCCGGCGCCGATGGGAAATGCGACCAAATGGGTTTCGAGGATCTCGATGGTGTTCTCTCCGGTGGCTCGCCGGGACAAGAACAGAGTATTTTTAGATTAATCATGGCTGGCGATGTAGTGGATCCGGGTTCGGAGTTTGTGGGTTTCGACATCGGTTCTGGATCCGACCCGGTTATTGATAATCCTAATCCACTCTTTGGATATGGCTTCCCTTTTCAAAACGCA |
| 36 | At rRNA 18S control(AT3g41768)  qRT-PCR primer sequences | Forward 5’ – CGTCCCTGCCCTTTGTACAC – 3’  Reverse 5’ – CGAACACTTCACCGGATCATT – 3’ |
| 37 | At SCL6-III (At3g60630) qRT-PCR primer sequences | Forward 5’ – ATGGGAAATGCGACCAAATG – 3’  Reverse 5’ – CGGAGAGAACACCATCGAGATC – 3’ |
| 38 | Taqman transgene expression primer and probe sequences (transcription terminator)- *A. thaliana* | Forward 5’ – TGATCACCTGTCGTACAGTATTTCTACA – 3’  Reverse 5’ – AGCCAGTGCTTGTTTTGTTTGA – 3’  FAM Probe 6FAM-TGATGTGTGATTTGTGAAGAA-MGB |
| 39 | Taqman expression primer and probe sequences (CTPA2 control) – *A. thaliana* | Forward 5’ – TGGTTGTTGTATAGGTCGGTGTAAC – 3’  Reverse 5’ – ATCCACAGAACGCCTCTTCAT – 3’  VIC Probe VIC-CATCCATTGCCAAAGTCGTTTCCGAA-TAMRA |
| 40 | At.miR171a,b,c northern probe sequence^14^ | 5’ - N222-cgtgatattggcgcggctcaatca – 3’ |
| 41 | At.miR159 northern control probe sequence^15^ | 5’ - N222-TAGAGCTCCCTTCAATCCAAA – 3’ |
|  |  |  |

1. miRNAs used for miRNA decoy proof of concept experiment

2. Decoy site in bold.

3. Decoy site embedded in the Zma.miR399 MIM transcript (item 5), replacing the endogenous

decoy site.

4. Gma-miRMON1 is an endogenous soy miRNA and mature sequence is found in item 1.

5. miR and miR* in bold.

6. Stop codon of gene is underlined.

7. Spacer is artificial and 75nt in length.

8. Sequence of interest is embedded in Gma-miRMON1 backbone.

9. miRNA target site in bold.

10. Decoy site placed at 3’ end of GUS+Spacer/Decoy reporter transcript (item 12).

11. Used in Supplementary Figure 3b.

12. a-miRGL1 is an engineered miRNA and mature sequence is found in item 2.

13. miR171 is a conserved family of miRNAs, and mature sequences are:

miR171a 5’- UGAUUGAGCCGCGCCAAUAUC-3’

miR171b,c 5’- uugagccgugccaauaucacg-3’

14. 25nt probe designed to detect mature At.miR171a, b and c linked to 222nt of non-native sequence so as to increase specific activity during DIG northern detection.

15. Probe designed to detect mature At.miR159 linked to 222nt of non-native sequence so as to increase specific activity during DIG northern detection.
