# Supplementary material for: Regulation of Gene Expression in Plants through miRNA Inactivation
Source: PLoS One. 2011 Jun 23;6(6):e21330. doi: 10.1371/journal.pone.0021330 (PMC3121747; doi:10.1371/journal.pone.0021330)
Supplement: Table S2 — Statistical analysis of qRT-PCR expression data of SCL6-III (At3g606030) in miR171_2B and miR171_3B plants versus wild type control. One-sized T-test Results from ANOVA. Results are from comparison of each event (homozygous (ho) or hemizygous (he)) versus the wild type control within each tissue, and adjusted p-values were computed from Dunnett's test. (DOC) [file pone.0021330.s006.doc]

**Table S2. Statistical analysis of qRT-PCR expression data of SCL6-III (At3g606030) in miR171_2B and miR171_3B plants versus wild type control.**

| **Tissue** | **Contrasts** | **Fold Change** | **log2(fold change)** | **Raw_P** | **Adj_P** |
| --- | --- | --- | --- | --- | --- |
| Flower | 171_3B Event 1-he | 1.80 | 0.85 | 3.08E-03 | 1.34E-02 |
| Flower | 171_3B Event 1-ho | 1.53 | 0.61 | 1.99E-02 | 7.39E-02 |
| Flower | 171_3B Event 2-ho | 1.74 | 0.80 | 4.51E-03 | 1.91E-02 |
| Flower | 171_2M Event 1-he | 1.30 | 0.38 | 9.55E-02 | 2.76E-01 |
| Flower | 171_2M Event 1-ho | 1.59 | 0.67 | 1.31E-02 | 5.06E-02 |
| Flower | 171_2M Event 2-ho | 1.11 | 0.15 | 3.01E-01 | 6.23E-01 |
| Leaf | 171_3B Event 1-he | 2.54 | 1.35 | 3.44E-05 | 1.78E-04 |
| Leaf | 171_3B Event 1-ho | 3.13 | 1.64 | 8.29E-06 | 4.39E-05 |
| Leaf | 171_3B Event 2-ho | 3.41 | 1.77 | 7.52E-07 | 4.09E-06 |
| Leaf | 171_2M Event 1-he | 1.42 | 0.51 | 4.17E-02 | 1.43E-01 |
| Leaf | 171_2M Event 1-ho | 1.82 | 0.87 | 2.55E-03 | 1.14E-02 |
| Leaf | 171_2M Event 2-ho | 1.90 | 0.93 | 1.53E-03 | 7.03E-03 |

One-sized T-test Results from ANOVA. Results are from comparison of each event (homozygous (ho) or hemizygous (he)) versus the wild-type control within each tissue, and adjusted p-values were computed from Dunnett’s test.
