# Supplementary material for: Regulation of Gene Expression in Plants through miRNA Inactivation
Source: PLoS One. 2011 Jun 23;6(6):e21330. doi: 10.1371/journal.pone.0021330 (PMC3121747; doi:10.1371/journal.pone.0021330)
Supplement: Table S3 — Statistical analysis of qRT-PCR expression data of SCL6-III (At3g606030) in miR171_3B transgenic decoy events versus miR171_2M transgenic decoy events. Two-sized T-test results from ANOVA. Results are from comparison of two constructs within each tissue type. No adjustments were done to the raw p-values. (DOC) [file pone.0021330.s007.doc]

**Table S3. Statistical analysis of qRT-PCR expression data of SCL6-III (At3g606030) in miR171_3B transgenic decoy events versus miR171_2M transgenic decoy events.**

| **Contrasts** | **fold change** | **log2(fold change)** | **Raw_P** |
| --- | --- | --- | --- |
| **171_3B vs 171_2M events in flower** | 1.28 | 0.35 | 2.29E-02 |
| **171_3B vs 171_2M events in leaf** | 1.76 | 0.82 | 1.36E-05 |

Two-sized T-test results from ANOVA. Results are from comparison of two constructs within each tissue type. No adjustments were done to the raw p-values.
